# Immunostimulatory CKb11 gene combined with immune checkpoint PD-1/PD-L1 blockade activates immune response and simultaneously overcomes the immunosuppression of cancer

**DOI:** 10.1016/j.bioactmat.2024.05.014

**Published:** 2024-05-23

**Authors:** Wen Nie, Yihong He, Xue Mi, Shi He, Jing Chen, Yunchu Zhang, Bilan Wang, Songping Zheng, Zhiyong Qian, Xiang Gao

**Affiliations:** a Department of Neurosurgery and Institute of Neurosurgery, State Key Laboratory of Biotherapy and Cancer Center, West China Hospital, West China Medical School, Sichuan University and Collaborative Innovation Center for Biotherapy, 610041, Chengdu, PR China; b Department of Pharmacy, West China Second University Hospital of Sichuan University, 610041, Chengdu, PR China

**Keywords:** Ovarian cancer, Immunogene therapy, CKb11, Immune checkpoint PD-1/PD-L1, Nanomedicine

## Abstract

Immunosuppression tumor microenvironment (TME) seriously impedes anti-tumor immune response, resulting in poor immunotherapy effect of cancer. This study develops a folate-modified delivery system to transport the plasmids encoding immune stimulatory chemokine CKb11 and PD-L1 inhibitors to tumor cells, resulting in high CKb11 secretion from tumor cells, successfully activating immune cells and increasing cytokine secretion to reshape the TME, and ultimately delaying tumor progression. The chemokine CKb11 enhances the effectiveness of tumor immunotherapy by increasing the infiltration of immune cells in TME. It can cause high expression of IFN-γ, which is a double-edged sword that inhibits tumor growth while causing an increase in the expression of PD-L1 on tumor cells. Therefore, combining CKb11 with PD-L1 inhibitors can counterbalance the suppressive impact of PD-L1 on anti-cancer defense, leading to a collaborative anti-tumor outcome. Thus, utilizing nanotechnology to achieve targeted delivery of immune stimulatory chemokines and immune checkpoint inhibitors to tumor sites, thereby reshaping immunosuppressive TME for cancer treatment, has great potential as an immunogene therapy in clinical applications.

## Highlights

• Immunogene therapy based on CKb11 and iPD-L1 regulates tumor immune microenvironment.
• Fa-modified nanomaterials target to tumor cells.
• Counterbalance the suppressive impact of PD-L1 on anti-cancer defense, leading to a collaborative anti-tumor outcome.
• Reshape immunosuppressive TME for cancer treatment.

## Graphical abstract

**Figure:**
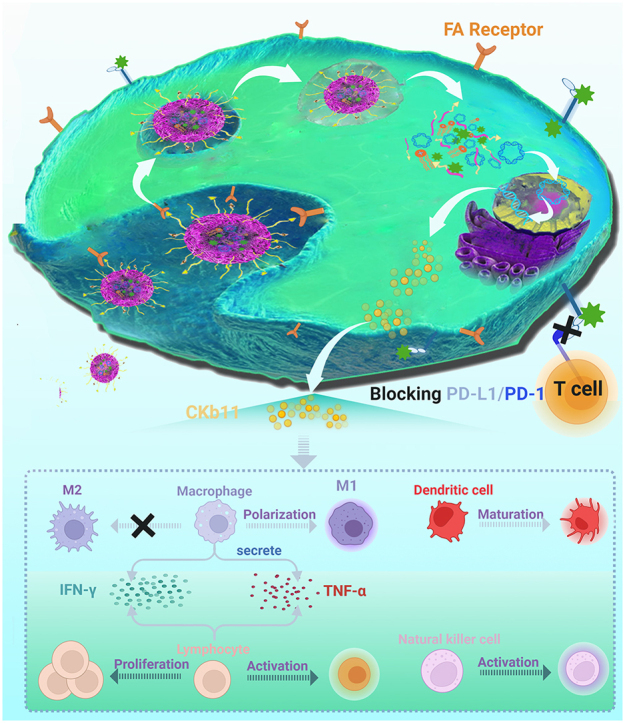

## 1 Introduction

Due to late detection and drug resistance, ovarian cancer has a high recurrence rate, a poor prognosis, and a high fatality rate [1]. At present, there is no effective early screening method for ovarian cancer, and most of the patients have lost the chance of radical surgery upon diagnosis [2]. The first-line treatment of ovarian cancer is mainly tumor cytoreductive surgery and postoperative chemotherapy [3,4]. The response rate to treatment of ovarian cancer patients is about 80 %, but about 75 % of the patients will relapse after 2–3 years of treatment, and many of them are resistant to chemotherapy. In addition, ovarian cancer is distinguished by immunosuppressive tumor microenvironment (TME) that suppresses the normal immune responses to fight against the tumor, leading to disappointing outcomes of immunotherapy [5]. Therefore, regulation of immunosuppressive TME will be a potential approach for ovarian cancer therapy.

Various immune modulators, including genes, vaccines, immune checkpoint blockers, cytokines, bispecific antibodies, and chimeric antigen receptor T cells have been developed for ovarian cancer treatments in the past few years [[6], [7], [8], [9]]. Moreover, the successful clinical application of multiple immunomodulating agents like interferon I (IFNs), interleukin 2 (IL-2) and gene drug antisense oligonucleotides have made immunotherapy strategy extremely attractive [[10], [11], [12]]. CKb11 is one of the C–C motif chemokine ligand of CCR7 expressed in the T-cell region of spleen, lymph nodes, lymphatic endothelium, and high endothelial venules [13]. It can induce infiltration of T cells, dendritic cells (DCs) and other lymphocytes through the concentration gradient and inhibit the apoptosis of mature DCs through the anti-apoptosis signaling pathway of CCR7 [14,15]. Studies have shown that CKb11 gene therapy has a significant anti-ovarian cancer effect and stimulates immune cells to secrete a large amount of IFN-γ while mediating immune activation and tumor eradication [16]. IFN-γ is reported to up-regulate the expression of programmed cell death-ligand 1(PD-L1) on tumor cells [17], thereby restricting immune cells from killing tumors. Therefore, combined therapy for achieving localized immunogene therapy in tumors proves to be a feasible clinical strategy.

Many tumors escape immune surveillance during immunotherapy. Numerous approaches have been suggested to tackle this problem and revive the immune response against tumors [[18], [19], [20]]. Among these, immune checkpoint inhibitors have demonstrated significant promise in the management of different types of cancers [21,22]. Programmed cell death-1 (PD-1), as an inhibitory receptor, is expressed on the surface of T cells, macrophages, NK cells, and B cells [[23], [24], [25]]. The conjugation of PD-1 and PD-L1 or Programmed cell death-2 (PD-L2) ligands can generate inhibitory signals, leading to the decrease of immune cell proliferation, cytotoxicity and cytokine production, and the suppression of the immune response [26]. Tumor cells express immunosuppressive checkpoint molecules (like PD-L1) to dampen the immune response, enabling them to evade and counteract immune detection. PD-1 and PD-L1 blockers can selectively block PD-L1 or PD-L2 mediated inhibitory immune response and promote the killing effect on tumors [27]. Despite the clinical advantages offered by immune checkpoint inhibitors, the effectiveness of individual inhibitors remains constrained as a result of the swift emergence of drug resistance. Several studies have explored the optimal combination strategy that targets multiple immune regulatory pathways to turn TME into a state of normal immunity, thus significantly increasing the effect of anti-tumor therapy [[28], [29], [30]]. Although combined immunotherapy has shown good anti-tumor effects and brought hope for many refractory tumors, the adverse reactions related to drug administration are still severe. In order to better deal with these problems and improve the safety of treatment, it is imperative to find more effective drug delivery methods.

In order to overcome the above obstacles, nanoscale carriers are designed to package therapeutic genes and small molecular inhibitors for good biocompatibility and tumor targeting, improving drug stability and reducing side effects [31,32]. Nanoparticles made from polymers have been attracting attention because of their unique properties, including easy modification performance, potential for powerful loading of multiple drugs, and reasonable biosafety [33,34]. They have broad application prospects in the fields of gene drugs, chemotherapy drugs, photosensitizers, and contrast agent delivery. FR is a membrane glycoprotein linked with glycosylphosphatidylinositol (GPI), which is over-expressed in ovarian cancer but under-expressed in normal tissues [35]. The surface of the gene-loaded nano-carrier is modified with folic acid (Fa) so that the nanostructure has specific cancer cell targeting, thus improving the efficiency of gene transfection. In addition, Fa has a relatively simple structure, simple and easy chemical bond with carrier and drug, no potential immunogenicity, low molecular weight and good stability [36].

**Scheme 1:**
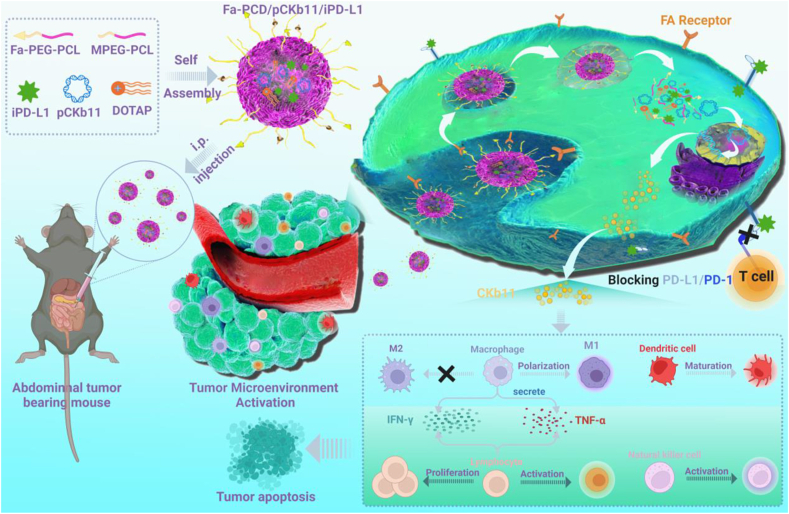
**Schematic illustration of the anti-tumor mechanisms of the combined immunogene therapy by Fa-PCD delivering pCKb11 and iPD-L1.** After administration, the delivery of pCKb11 by Fa-PCD achieved tumor-specific secretion of CKb11. The delivery of iPD-L1 by Fa-PCD blocked the immunosuppressive role of PD-L1 overexpression in tumor cells induced by higher IFN-γ concentration. The codelivery of pCKb11 and iPD-L1 by Fa-PCD significantly reshapes the immunosuppressive TME, inducing proliferation and activation of lymphocytes, macrophage repolarization, and DC maturation in TME, and inhibits tumor progression.

Based on the above, we suggest a strategy for immunotherapy targeting the folate receptor, which involves the combination of plasmid DNA encoding the chemokine CKb11, known for its immunostimulatory properties, and PD-L1 inhibitor (BMS-1), a small molecule inhibitor that disrupts the interaction between PD-1 and PD-L1. This strategy aims to modulate the immunosuppressive TME and stimulate the immune response, ultimately leading to the inhibition of ovarian cancer progression (Scheme 1). This immunostimulatory therapy, in conjunction with immune checkpoint inhibitors, can simultaneously target multiple immune regulatory pathways to synergize the anti-tumor immune response, leading to a substantial improvement in therapeutic outcomes. It offers a promising avenue for developing safe and effective immunotherapy strategies.

## 2 Materials and methods

### 2.1 Preparation and encapsulation of nanocomposites

For targeted nanoparticles Fa-PCD/pCKb11/iPD-L1，first, Fa-PEG-PCL (15 mg), MPEG-PCL (70 mg), DOTAP (10 mg) and BMS-1 (bought from MedChemExpress) were added to a flask and dissolved in acetone. Then, evaporate the acetone under negative pressure. Positively charged nanoparticles loading BMS-1 were prepared by adding 5 mL of 5 % glucose to the flask. Then, mix the nanoparticles and pDNA in equal volume for 20 min when using. Pvax (Invitrogen, US) vectors were used to construct the CKb11-encoding plasmids (pCKb11). As a control, pvax was used. All the plasmids were extracted by the EndoFree Plasmid Giga kit (Qiagen, Hilden, Germany). The non-targeted nanoparticles PCD/pCKb11/iPD-L1 were prepared without Fa-PEG-PCL by the same method described above.

### 2.2 Agarose gel retardation electrophoresis experiment

Mixing pCKb11 with Fa-PCD at different mass ratios for 20 min was performed. The prepared mixture and DNA ladder were added to the corresponding wells of 1 % agarose gel prepared in advance. Then, the electrophoresis was performed at 120 V and terminated when the loading buffer band on the gel was completely displayed. Finally, the gel was taken out and put into a gel imager (Bio-rad, US) for imaging. For a dilution stability test, pDNA and Fa-PCD/pDNA were respectively diluted at a serial dilution (0, 2, 4, 8, 16, 32, 64, 128, 256, 512 fold) and then performed agarose gel electrophoresis retardation assay. For serum stability test, pDNA and Fa-PCD/pDNA nanoparticles were respectively incubated in 1640 media with DNase I (1U/mL) for different time. After 0, 30, 45, 60, 90, 150, 180, 360 min, samples were collected for agarose gel electrophoresis experiments. Also, the Fa-PCD/pDNA nanoparticles were incubated in serum (fresh 1640 media with 10%FBS) for different time. After 0, 2, 6, 12, 24, 48 h, samples were collected for agarose gel electrophoresis experiments.

### 2.3 Plasmid tracing in vitro

ID8 cells were cultured in plates overnight. pCKb11 and YOYO-1 (Invitrogen, US) were mixed and incubated in DMEM double-free medium (Gibico, US) for 20 min to prepare solution A. Then the solution A was slowly and gently added to the transfection reagent for mixture and incubated for 20 min to make solution B. Then Lyso-tracker (Thermofisher, US) with a working concentration of 70 μM and Hoechst 33342 (Thermofisher, US) reagent with a working concentration of 1 μg/mL were added respectively to solution B to make solution C. After the DMEM complete medium was replaced by the prepared solution C, the culture plates were placed under a Zeiss laser confocal microscope (Carl Zeiss, Germany) to observe the plasmid uptake and intracellular delivery process in real time.

### 2.4 Cell lines and experimental animals

The mouse ovarian cancer cell line ID8 used in the experiment was provided by the State Key Laboratory of Biotherapy (SKLB), Sichuan University. The cell culture medium was DMEM high-glucose medium (Gibico, US) containing 10% fetal bovine serum (Gibico, US) and 100 μg/mL penicillin and streptomycin (Thermofisher, US). Female C57 mice (6–8 weeks) were purchased from Beijing Huafukang Biotechnology Co., Ltd, and housed in the specific pathogen-free (SPF) animal room of West China Science and Technology Park, Sichuan University. Strictly following the good animal ethics, all animal experiments were carried out in accordance with the regulations of the Animal Experiment Ethics Committee of the State Key Laboratory of Biotherapy, Sichuan University.

### 2.5 Evaluation of pEGFP transfection efficiency

pEGFP, Fa-PCD, and MPCD were dissolved in serum-free DMEM medium respectively for 5 min. Next, the solution containing pEGFP was gradually introduced into the solution containing Fa-PCD and MPCD, and allowed to stand for 20 min to form the Fa-PCD/pEGFP or PCD/pEGFP complex. The nanocomposite-containing medium was applied to cells and cultured in the incubator at 37°C for 4 h of transfection. After that, the transfection reagents were replaced with fresh complete medium for 48 h. Finally, the transfection and expression of Fa-PCD/pEGFP or PCD/pEGFP were observed under an inverted fluorescence microscope, and the transfection efficiency was determined by flow cytometry.

### 2.6 Electron microscope observation and particle size, potential analysis and reduction responsiveness determination of nanocomposites

First, Fa-PCD/pCKb11/iPD-L1 and PCD/pCKb11/iPD-L1 were prepared, and then a small drop of the liquid was dropped on the copper grid. After soaking the copper grid, the liquid was completely absorbed by the qualitative filter paper. After that, phosphotungstic acid was added dropwise for negative staining for 3 min. Subsequently, the dye solution was absorbed by qualitative filter paper with the copper grid placed on it, and dried at room temperature. Then the morphological characteristics were observed under a transmission electron microscope (TEM; JEOL, Japan). Second, after properly diluting the nanocomposite, about 1 mL of the liquid was put into the particle size cup and sent to the particle size analyzer. The parameters were properly set to detect the mean particle size and *zeta* potential of the nanocomposite. Also, Fa-PCD (vehicle) and Fa-PCD/iPD-L1 (iPD-L1) were experienced the mean particle size and *zeta* potential detection. For dilution stability test, the Fa-PCD/pDNA experienced a serial dilution and then its particle size was tested. For serum stability test, the Fa-PCD/pDNA nanoparticles were incubated in 1640 media with DNase I (1U/mL) or in serum (fresh 1640 media with 10%FBS) for different time, and samples were collected for size measurement.

### 2.7 In vitro assays for the effect of different regimens on the viability of cells

The tumor cells and the successfully isolated lymphocytes were respectively placed in a 96-well plate, and different regimens were added for 24 h. Isolated lymphocytes were stimulated with supernatants from transfected ovarian cancer cells. Then, the Cell Counting Kit-8 (CCK8) Assay Kit (Beyotime, China) was used to detect cell activity according to the instruction.

### 2.8 Assays for the effect of different regimens on macrophages and DC

The successfully induced BMDMs were plated and incubated with the supernatants of tumor cells transfected by different groups (Untreated, Vehicle, Fa-PCD/pvax, iPD-L1, Fa-PCD/pCKb11, PCD/pCKb11/iPD-L1 and Fa-PCD/pCKb11/iPD-L1) for 48 h. The macrophages were digested and collected to prepare samples to further investigate related molecular pathways in macrophage polarization by FCM, Western blot analysis and RT-PCR. Supernatants of tumor cells transfected by different groups (Untreated, Vehicle, Fa-PCD/pvax, iPD-L1, Fa-PCD/pCKb11, PCD/pCKb11/iPD-L1 and Fa-PCD/pCKb11/iPD-L1) were collected to stimulate successfully induced BMDM cells from wild-type mice. After 48-h stimulation, the macrophages were collected, centrifuged and resuspended in PBS staining solution containing antibodies (CD45, CD11b, F4/80 and CD206) for FCM. After incubation, wash and resuspension, the cells were analyzed on a flow cytometer (ACEA NovoCyte, US). The total proteins of the treated BMDM cells in each group were also collected, and several macrophage phenotype-related proteins were detected by Western blot analysis including iNOS, STAT1, P-STAT1, P–P65, p65, IRF4, PPAR-r, IRF3, and P-IRF-3. Furthermore, the RNA of the BMDM cells after treatment in each group was isolated by a RNAsimple Total RNA Kit (TIANGEN, China), and the RNA concentration of TNF-α, IL-12, Citta, CXCL10, IFN-r, CXCL19, IRF5, NOS2, IL-6, MRC-1, YM-1, and Fizz1 was detected using RT-PCR. Sequences of PCR primers were shown in Table S1.The successfully induced DCs were plated and incubated with the supernatants of tumor cells transfected by different groups (Untreated, Vehicle, Fa-PCD/pvax, iPD-L1, Fa-PCD/pCKb11, PCD/pCKb11/iPD-L1 and Fa-PCD/pCKb11/iPD-L1) for 48 h. The DCs were digested and collected to prepare samples for FCM by antibodies (CD11c, CD80, CD86 and MHCII). Information of flow cytometry antibodies was shown in Table S2.

### 2.9 Assays for the effect of different regimens on lymphocytes

To determine lymphocyte proliferation, supernatants of tumor cells transfected by different groups (Untreated, Vehicle, Fa-PCD/pvax, iPD-L1, Fa-PCD/pCKb11, PCD/pCKb11/iPD-L1 and Fa-PCD/pCKb11/iPD-L1) were collected to stimulate lymphocytes isolated from wild-type mice. After stimulation, the cells were collected and labeled with CD8, CD4, CD69 and INF-γ.

For determining the secretion of antitumor cytokines, successfully isolated lymphocytes were added to tumor cells treated with each reagent, and after co-cultivation for 24 h, the supernatants in each group were collected, and the secretion of antitumor cytokine IFN-γ and TNF-α was determined respectively following the standard instructions of the Elisa kit (Thermo Scientific, US).

### 2.10 Labeling tumor cell killing assay

HBSS solution containing CFSE dye (1:5000) was used to incubate ID8 cells for 15 min. After staining, cells were resuspended and plated in 12-well plates. After cell adhesion, CFSE-prelabeled ID8 cells were transfected with reagents of each group. After 24 h, successfully isolated lymphocytes were added, and the co-culture system was held for 24 h. The co-incubated cells were observed and taken pictures under a microscope (Olympus, Japan). Then, the cells were also stained with binding buffer containing Annexin V-PE dye for 15 min. Then, FCM was used to evaluate the percentage of apoptotic cells.

### 2.11 Tumor models establishment

For intraperitoneal metastasis of ovarian cancer mouse model, 200 μL free medium containing 2 × 10^6^ ID8 cells was intraperitoneally inoculated into Balb/C female mice. Upon inoculation of one week, the mice with tumors were randomly divided into the following groups: GS, Vehicle, Fa-PCD/pvax, iPD-L1, Fa-PCD/pCKb11, PCD/pCKb11/iPD-L1 and Fa-PCD/pCKb11/iPD-L1 (dose: 10 μg of plasmid per mouse, iPD-L1 10 mg/kg). The administration method was intraperitoneal injections, which were given 12 times, twice a week, and the weight change of the mice was monitored and recorded once a week.

### 2.12 Therapeutic efficacy evaluation

After the treatment was finished, the mice were euthanized on the following day, and the tumor nodules, ascites, vital organs and blood were gathered. Mice and the removed tumors were weighed. Corresponding cells were collected to perform FCM analysis of the TME. Supernatants of ascites, tumor tissue lysis product and serum were analyzed by Elisa assay. Tumors and organs were fixed, embedded, and H&E or immunohistochemical stained for histopathological examination. Tumor cell proliferation was determined by Ki67 staining, and microvessels was evaluated by CD31 staining according to the immunohistochemistry staining protocols. Five randomized fields were used to count and analyze the number of vessels positive for CD31 and the proportion of cells positive for Ki67.

### 2.13 Statistical analysis

GraphPad PRISM Version 8.0 software was utilized to analyze all of the data. The comparison between two data groups was analyzed by *t*-test, and the comparison between multiple data groups was analyzed by ANOVA test. The mean ± standard error of measurement was used to express all values. When p is less than 0.05, the difference is significant. p* is less than 0.05, p** is less than 0.01, p*** is less than 0.001, p**** is less than 0.0001, n. s. means no significant.

## 3 Results

### 3.1 Preparation and characteristics of Fa-PCD/pCKb11/iPD-L1

**Fig. 1:**
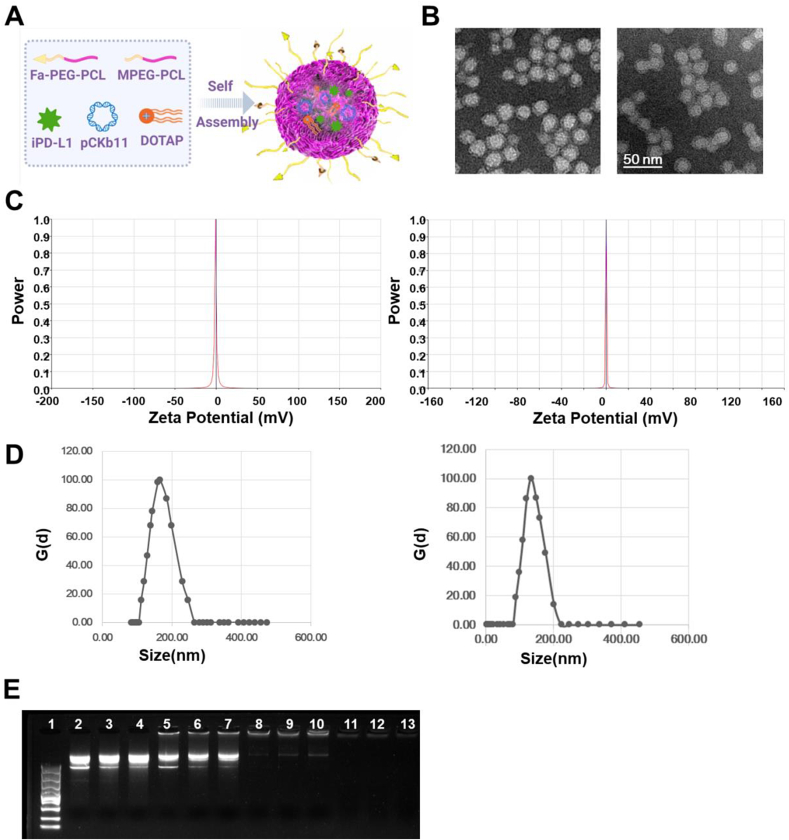
**Characteristics of the nanocomposites**. (A) A model of self-assembled nanocomposite Fa-PCD/pCKb11/iPD-L1. (B) TEM of PCD/pCkb11/iPD-L1 (left) and Fa-PCD/pCkb11/iPD-L1 (right). (C) *Zeta* potential of PCD/pCkb11/iPD-L1 (left) and Fa-PCD/pCkb11/iPD-L1 (right). (D) Sizes of PCD/pCkb11/iPD-L1 (left) and Fa-PCD/pCkb11/iPD-L1 (right). (E) Agarose gel electrophoresis of Fa-PCD/pCkb11/iPD-L1. Lane 1 marker; 2–4, naked pCKb11; lane 5–13: different mass ratios of composites with pCKb11 (pCKb11: Fa-PCD, lane 5–7, 1:25; lane 8–10, 1:50; and lane 11–13, 1:100), (scale bar, 50 nm; n = 3).

The synthesis steps of MPEG-PCL and Fa-PEG-PCL were listed in Figs. S1 and S2, whose structures were further confirmed by the ^1^H NMR (Figs. S3 and S4). Fig. S5A. Fa-PEG-PCL, MPEG-PCL, DOTAP, iPD-L1 and pCKb11 were prepared and self-assembled to form a core-shell structure, Fa-PCD/pCKb11/iPD-L1 (Fig. 1A). In the core of the Fa-PCD/pCKb11/iPD-L1 nanocomposites, the positive charges of DOTAP in the center of the Fa-PCD/pCKb11/iPD-L1 nanocomposites can efficiently attract pCKb11 through electrostatic interaction. Under TEM, the Fa-PCD/pCKb11/iPD-L1 and PCD/pCKb11/iPD-L1 nanocomposites were observed to be spherical (Fig. 1B). The *zeta*-potential of Fa-PCD/pCKb11/iPD-L1 and PCD/pCKb11/iPD-L1 were almost electrically neutral (Fig. 1C), the mean sizes of them were respectively 173.96 nm and 171.84 nm (Fig. 1D). The *zeta*-potential and mean sizes of control nanoparticles was shown in Figs. S5A-D). In addition, we performed the agarose gel electrophoresis test to determine the ability of Fa-PCD nanoparticles to load genes (Fig. 1E). The naked pCKb11 were shown as bright ladders (columns 2 to 4), while the cumulative retardation of pCKb11 was noted using weight ratios (pCKb11: Fa-PCD) of 1:25 (columns 5 to 7) and 1:50 (columns 8 to 10). Under the mass ratio of 1:100 (columns 11 to 13), no bright ladders were observed, suggesting that pCKb11 was completely encapsulated by Fa-PCD. The nuclease degradation resistance and serum stability of Fa-PCD nanoparticles have performed by agarose gel electrophoresis experiments and particle size assay. As shown in Fig. S6A, pDNA was degraded after 30 min, while Fa-PCD/pDNA was stable after 360 min in 1640 media with DNase I (1U/mL). Also, size variation of Fa-PCD/pDNA at different time is little, which suggests Fa-PCD nanoparticles can resist nuclease degradation (Figs. S6A and B). In addition, pDNA and Fa-PCD/pDNA nanoparticles were incubated in serum (fresh 1640 media with 10%FBS) for different time. The results showed that no degradation and little size variation of F-PCD/pDNA was observed after 48 h (Figs. S6C–E). Thus, Fa-PCD nanoparticles are resistant to nuclease and stable in serum. To further evaluate dilution stability, the diluted Fa-PCD/pDNA was experienced an agarose gel electrophoresis test and particle size assay. The results showed that no free pDNA bands were detected in diluted Fa-PCD/pDNA, demonstrating that Fa-PCD/pDNA nanoparticles were very stable and kept their initial size and size distribution undergoing up to 64-fold dilution (Fig. S7A). In addition, size variation of Fa-PCD/pDNA after a serial dilution is little, especially when the dilution ratio is less than 128-fold (Fig. S7B). These results suggested that the condensation of pDNA and well-defined nanoparticles of Fa-PCD/pDNA are maintained during a high-dilution process.

### 3.2 Distribution and transfection efficiency of Fa-PCD/pEGFP in ovarian cancer cells

**Fig. 2:**
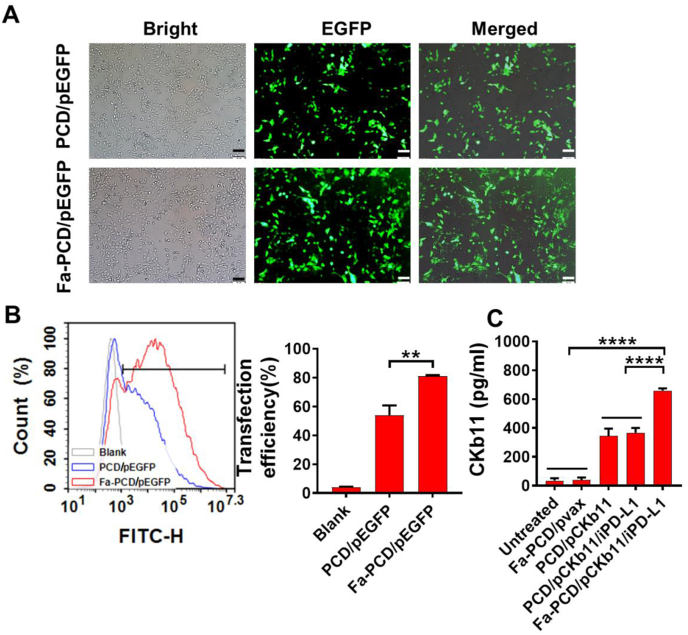
**The transfection efficiency of the nanoparticles**. (A) Fluorescent images of ID8 cells treated with Fa-PCD/pEGFP and PCD/pEGFP for 48 h. (B) Transfection efficiency by FCM. (C) Quantification of CKb11 released by ID8 cells by Elisa. (n = 3, **p < 0.01, ****p < 0.0001; One-way ANOVA).

The transfection efficiency of Fa-PCD was assessed by transfecting with pEGFP to ovarian cancer cells (ID8). As shown in Fig. 2, ovarian cancer cells transfected with Fa-PCD/pEGFP exhibited significantly higher EGFP expression compared to those transfected with PCD/pEGFP (Fig. 2A). The FCM analysis further validated the result (Fig. 2B), with about 85 % transfection efficiency for Fa-PCD/pEGFP and 50 % for PCD/pEGFP. Further, after Fa-PCD/pCKb11/iPD-L1 transfection into ovarian cancer cells, the quantity of CKb11 expression in cell supernatants was detected by Elisa. As shown in Fig. 2C, the secretion of CKb11 was enhanced by Fa-PCD-delivered plasmid in comparison to the non-targeted system. The intracellular distribution of Fa-PCD/pCKb11 in ovarian cancer cells was detected to verify the endosomal escaping capability of the gene vector. After being administered by Fa-PCD, the YOYO-1 tagged pCKb11 was initially found in the cytoplasm co-located with Lyso-tracker-labeled endolysosomes, according to the continuous tracing (Fig. S8A). Finally, the Fa-PCD/pCKb11 gradually escaped from endosomes and entered the nucleus completely after 5 h. The fluorescence intensities and positions of pCKb11 and nucleus demonstrated the ability of Fa-PCD to escape from endolysosomes, as shown in the overlay (Figs. S8B and C). These results prove that Fa-PCD efficiently delivers pCKb11 to the nucleus, resulting in successful expression.

### 3.3 Fa-PCD/pCKb11/iPD-L1 significantly activates lymphocytes and promotes apoptosis of ovarian cancer cells in vitro

**Fig. 3:**
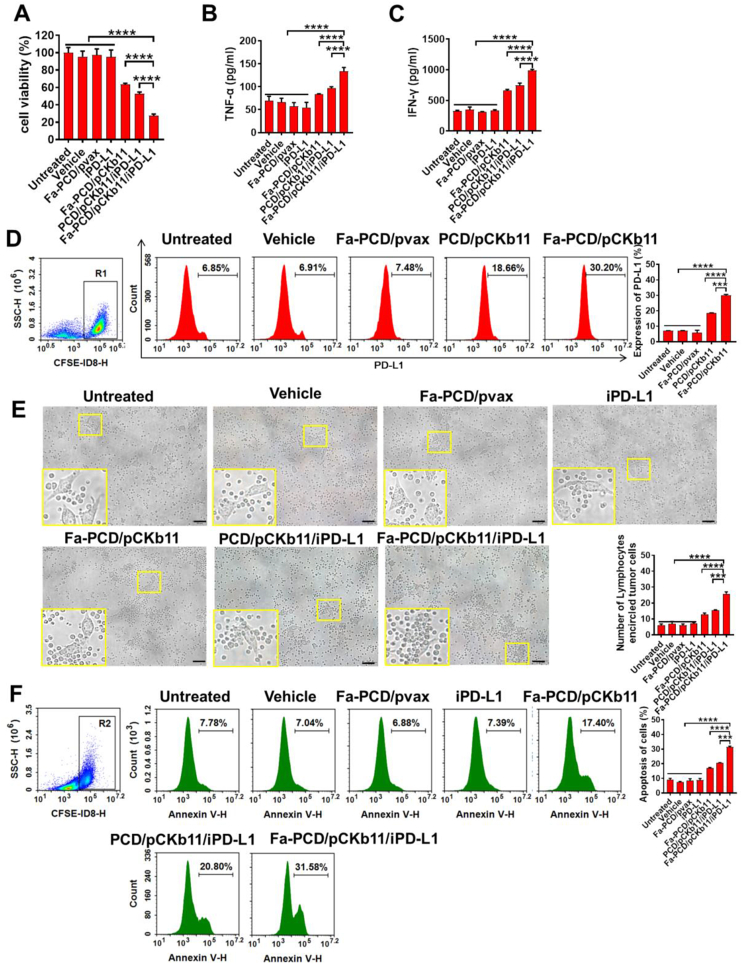
**The treatment of Fa-PCD/pCKb11/iPD-L1 enhanced cytokines secretion from lymphocytes to promote apoptosis of tumor cells**. (A) The cell viability of ID8 cells was evaluated by CCK8 assay. (B–F) Co-incubation of CFSE-labeling ID8 cells treated with different nanocomposites and lymphocytes. TNF-α (B) and IFN-γ (C) secreted from lymphocytes by Elisa. (D) PD-L1 expression of CFSE-labeling ID8 cells by flow cytometry. (E) Representative images of ID8 cells co-cultured with lymphocytes at a ratio of 1:20 (ID8 cells: lymphocytes). Statistical results showed an average number of lymphocytes surrounding a tumor cell. (F) The apoptosis of CFSE-labeling ID8 cells was assessed by FCM. (n = 3, ***p < 0.001, ****p < 0.0001; One-way ANOVA; scale bar, 50 μm).

In vitro, we examined the impact of Fa-PCD/pCKb11/iPD-L1 on immune cells, especially T cells, DCs and macrophages that exhibited significant CCR7 expression. The ID8 cells were subjected to different treatments for 48 h. Subsequently, the supernatants obtained from the transfected ovarian cancer cells were gathered to induce stimulation in the isolated lymphocytes. Afterward, the liquids from the activated lymphocytes were gathered to cultivate untreated ID8 cells. Notably, supernatants of lymphocytes in the Fa-PCD/pCKb11/iPD-L1 group significantly inhibited the ovarian cancer cell viability (Fig. 3A). Fig. 3B and C demonstrate that the incubating lymphocytes with the supernatants from ID8 cell transfected with Fa-PCD/pCKb11/iPD-L1 stimulated the release of TNF-α and IFN-γ, which are proinflammatory cytokines critical for elimination of tumor cells. Furthermore, the PD-L1 expression in ID8 cells was up-regulated after pCKb11 transfection and incubation with lymphocytes (Fig. 3D), which proved that the treatment scheme of CKb11 gene therapy combined with iPD-L1 is rational. The ID8 cells transfected with pCKb11 for 24 h were co-cultured with isolated lymphocytes for 48 h, and it was found that the ID8 cells treated with Fa-PCD/pCKb11/iPD-L1 were surrounded by abundant lymphocytes (Fig. 3E). The co-culture with lymphocytes caused ID8 cells apoptosis in pCKb11 transfected groups, combining with iPD-L1 further promoted tumor cell apoptosis, especially using the Fa-targeted delivery system (Fig. 3F).

**Fig. 4:**
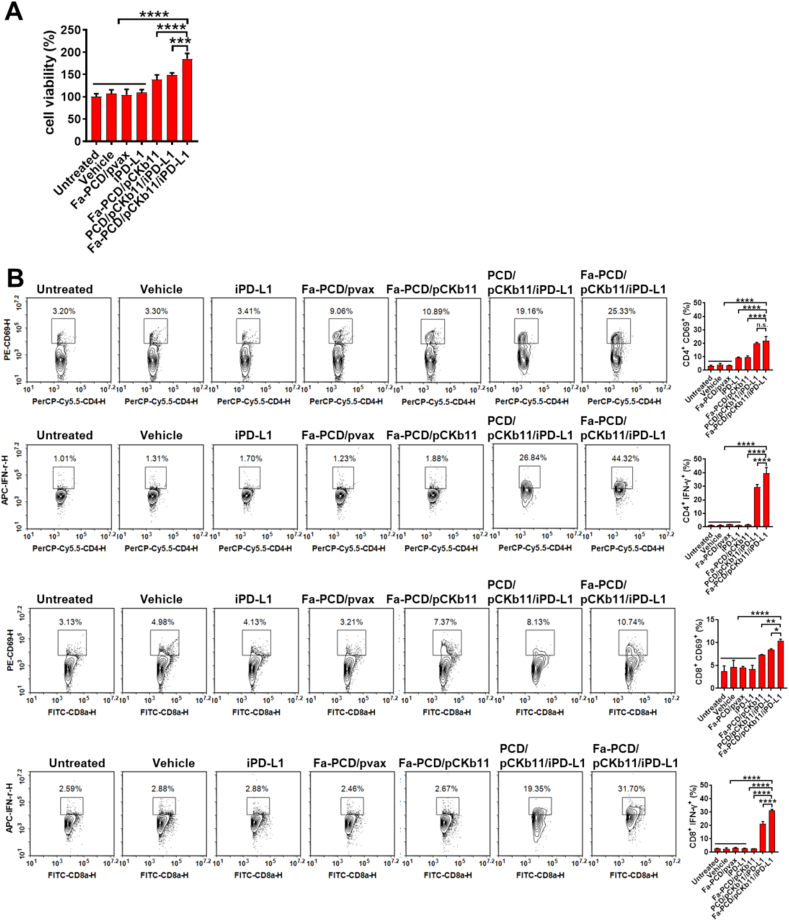
**Fa-PCD/pCKb11/iPD-L1 treatment promotes proliferation and activation of lymphocytes**. ID8 cells were treated with different nanocomposites for 48 h. Then the cell supernatants were collected for treating lymphocytes. (A) Cell viability of lymphocytes assessed by CCK8. (B) Activated T lymphocytes detected by FCM, which were defined as CD8^+^CD69^+^, CD8^+^IFN-γ^+^, CD4^+^CD69^+^, CD4^+^IFN-γ^+^ lymphocytes. (n = 3, *p < 0.05, **p < 0.01, ****p < 0.0001, n. s. , no significance; One-way ANOVA).

In addition, the viability of lymphocytes treated with supernatants from the Fa-PCD/pCKb11/iPD-L1 group was higher than any other groups (Fig. 4A). CD69 and IFN-γ are usually used as markers of T-cell activation. As shown in Fig. 4B, CD4^+^ and CD8^+^ T cells were activated when exposed to the supernatants from transfected ID8 cells in the Fa-PCD/pCKb11/iPD-L1 and PCD/pCKb11/iPD-L1 group with increased percentages of CD69^+^ and IFN-γ^+^ T lymphocytes, especially in the Fa-PCD/pCKb11/iPD-L1 group. Thus, it is indicated that the successful release of CKb11 by cancer cells and the perfect blockade of PD-1/PD-L1 by iPD-L1 effectively activate lymphocytes and promote them to secret proinflammatory cytokines.

### *3.4* Fa-PCD/pCKb11/iPD-L1 *regulates macrophage polarization*

**Fig. 5:**
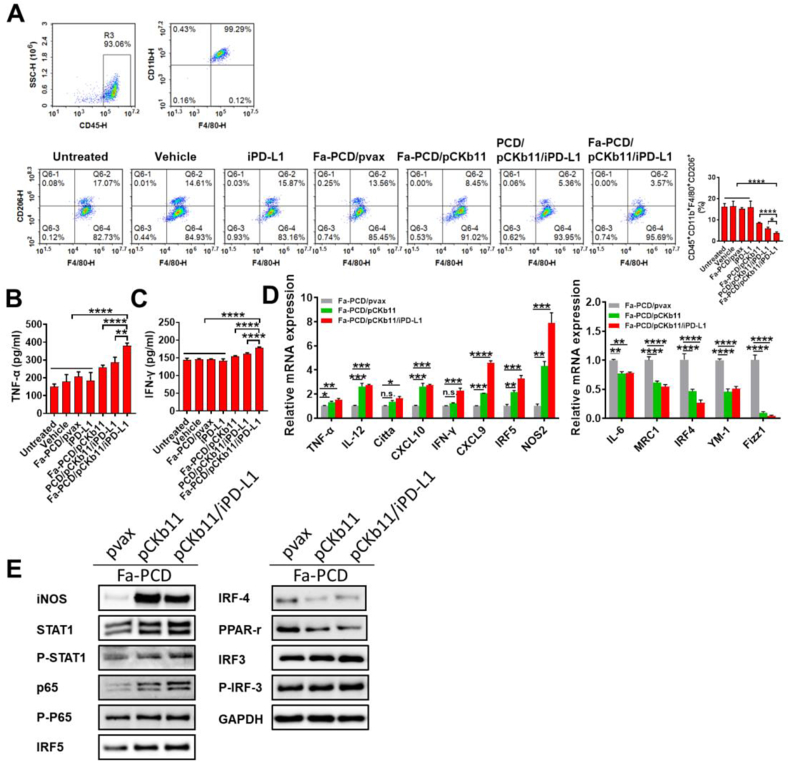
**Fa-PCD/pCKb11/iPD-L1 treatment inhibits M2-polarization. ID8 cells were treated with different nanocomposites for 48h**. The cell supernatants were collected to treat macrophages for 48 h. Then the supernatants from macrophages were collected for Elisa. The macrophages were collected for flow cytometry, PCR and WB. (A) Polarization status of macrophages was assessed by flow cytometry. Polarized M2 cells were defined as CD45^+^CD11b^+^F480^+^ CD206^+^ macrophages. (B) TNF-α and (C) IFN-γ expression in supernatant from macrophages was detected by Elisa. (D) Relative mRNA expression of macrophage-related genes by RT-PCR. (E) Expression of macrophages-related proteins by WB. (n = 3, *p < 0.05, **p < 0.01, ***p < 0.001, ****p < 0.0001; One-way ANOVA).

Macrophages are characterized by two distinct polarization statuses according to their activation patterns and functions, which are the pro-inflammatory M1 phenotype with an anti-tumor effect and the immune suppressive M2 phenotype with a tumor-promoting function. The majority of tumor-associated macrophages (TAMs) are the M2 phenotype exhibiting immunosuppressive profiles. Thus, reducing the proportion of M2-polarized macrophages in TME plays a significant role in cancer immunotherapy. In this study, the supernatants of ID8 cells treated differently for 48 h were collected to stimulate isolated macrophages. As shown in Fig. 5A, the percentages of M2-polarized cells (CD45^+^CD11b^+^F4/80^+^CD206^+^ cells), especially in the Fa-PCD/pCKb11/iPD-L1 group was decreased. Meanwhile, macrophages in the Fa-PCD/pCKb11/iPD-L1 group secreted more IFN-γ and TNF-α (Fig. 5B and C). Furthermore, using real-time polymerase chain reaction (RT-PCR), we assessed the expression levels of a number of genes that are likely to be involved in macrophage inflammation or immunosuppression. As shown in Fig. 5D, the inflammation-related gene expression of TNF-α, IL-12, Citta, CXCL10, IFN-γ, CXCL9, IRF5 and NOS2 were obviously up-regulated, while the immunosuppressive-related genes YM-1, IL-6, IRF4, MRC1 and Fizz1 were significantly down-regulated. To promote M1 polarization of macrophages, STAT1 activation is extremely important. The STAT1 pathway was markedly activated in macrophages stimulated with supernatants from Fa-PCD/pCKb11/iPD-L1 transfected ID8 cells, as demonstrated in Fig. 5E. This activation was accompanied by an increase in iNOS and p65 protein levels as well as P-STAT1, P-IRF3, and a decrease in IRF-4 and PPAR-r protein levels. It can be seen from the above that the M2 pathway is inhibited while the M1 pathway is also activated, thereby promoting tumor growth.

### 3.5 Fa-PCD/pCKb11/iPD-L1 promotes maturation of DCs

**Fig. 6:**
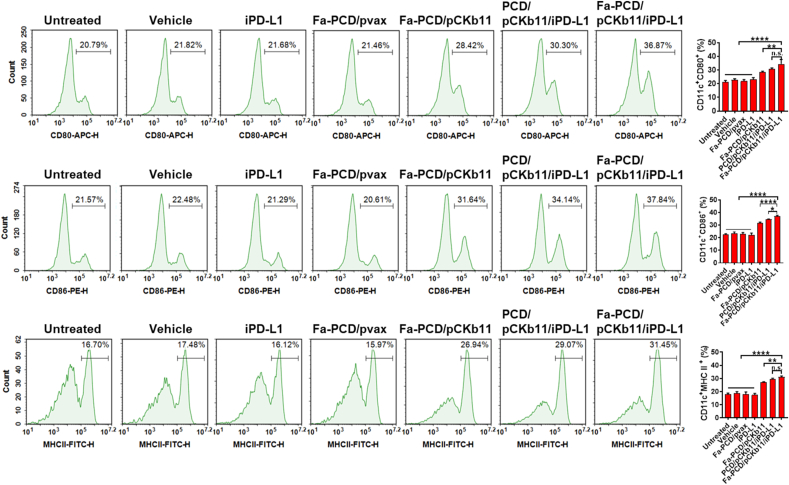
**Mature DCs are increased after treatment of Fa-PCD/pCKb11/iPD-L1**. ID8 cells were treated with different nanocomposites for 48 h. The cell supernatants were collected to treat DCs for 48 h. Then the DCs were collected and detected by FCM. CD11c^+^CD80^+^, CD11c^+^CD86^+^ and CD11c^+^MHC II^+^ were defined as mature DCs. (n = 3, **p < 0.01, ****p < 0.0001, n. s. , no significance; One-way ANOVA).

In addition to the above immune cells, mature DCs were detected by labeling cell surface antibodies, CD80, CD86 and MHCII, which play the role of antigen presentation in immune response. As shown in Fig. 6, the supernatant from ID8 cells with successful pCKb11 transcription promoted the maturation of DCs. The addition of PD-L1 inhibitors further enhanced its role in promoting DC maturation. In addition, the targeted delivery system group had more matured DCs than the non-targeted. Taken together, it is proven that the Fa-PCD/pCKb11/iPD-L1 could effectively stimulate the maturation of DCs.

### 3.6 Fa-PCD/pCKb11/iPD-L1 suppressed the progression of ovarian cancer

**Fig. 7:**
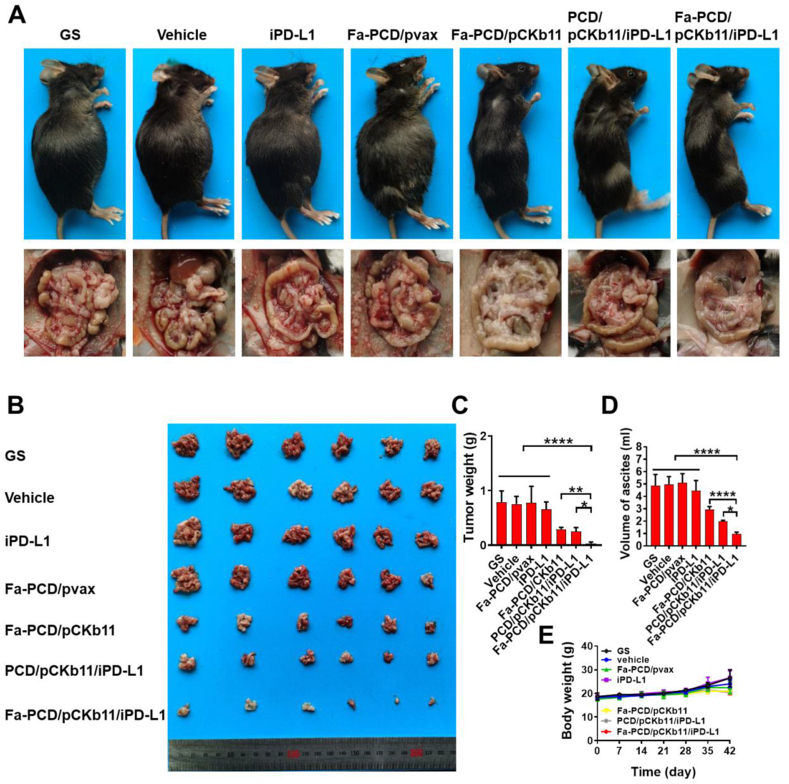
**Fa-PCD/pCKb11/iPD-L1 inhibited tumor growth**. The abdominal ID8 tumor-bearing mice were intraperitoneally injected according to their groups for 42 days. Then mice were sacrificed and tumors were collected. (A) Representative images of tumor-bearing mice and their corresponding abdominal cavity. (B) Images of tumors. (C) Tumor weight. (D) Volume of ascites. (E) Body weight. (n = 6, *p < 0.05, **p < 0.01, ****p < 0.0001; One-way ANOVA).

**Fig. 8:**
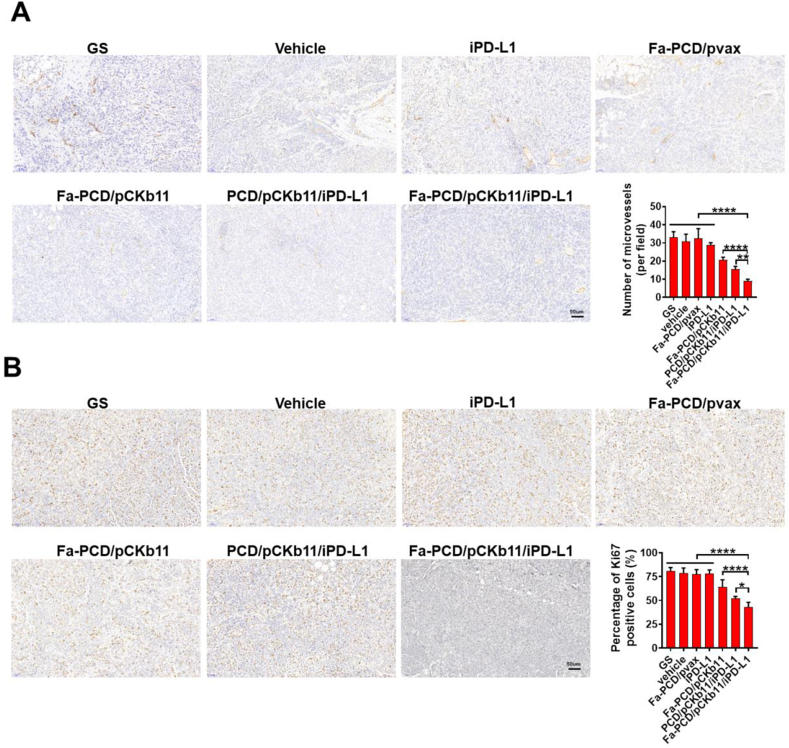
**Fa-PCD/pCKb11/iPD-L1 inhibited tumor proliferation and angiogenesis**. (A) CD31 and (B) Ki67 immunohistochemistry of ID8 tumors. (scale bar, 50 μm, n = 5, *p < 0.05, **p < 0.01, ****p < 0.0001; One-way ANOVA).

Mouse ovarian cancer intraperitoneal implantation model was established to evaluate the different composites’ anti-tumor effects. According to Fig. 7A and B, the mice in groups of pCKb11 transcription had a smaller abdominal circumference and fewer abdominal tumor nodules. In particular, the Fa-PCD/pCKb11/iPD-L1 treatment exhibited the most robust anti-tumor effect with the lowest average weight of abdominal tumor and volume of ascites, and no notable alteration in body weight when compared to the remaining groups (Fig. 7C–E). Moreover, the tumor vessels were examined using CD31 staining, while the in vivo proliferation of tumor cells was assessed through Ki-67 staining (Fig. 8A and B). The Fa-PCD/pCKb11/iPD-L1 treatment had the strongest anti-vascular effect with the least amount of microvessels, as well as the most potent anti-tumor proliferation ability with the lowest percentage of Ki-67 positive cells. Thus, Fa-PCD/pCKb11/iPD-L1 therapy could effectively suppress ovarian cancer progression by decreasing tumor angiogenesis and suppressing cellular proliferation.

### 3.7 Fa-PCD/pCKb11/iPD-L1 reshaped the immunosuppressive TME in vivo

**Fig. 9:**
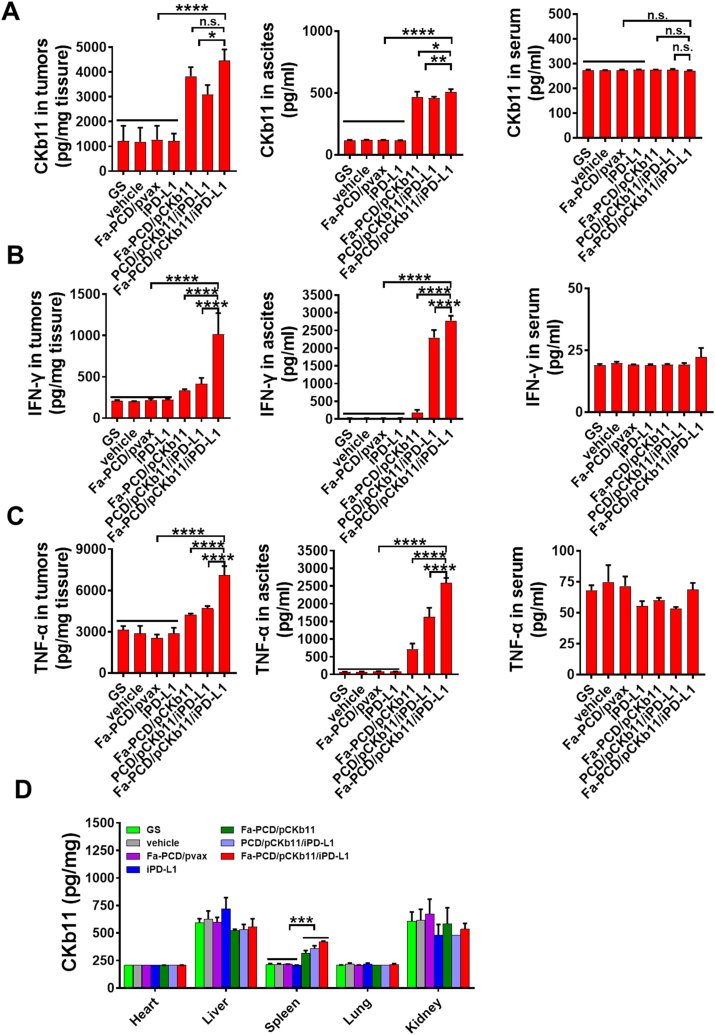
**Fa-PCD/pCKb11/iPD-L1 increased CKb11, IFN-γ and TNF-α expression in vivo**. Mice-bearing tumors were treated for 42 days. Then mice were sacrificed and their blood, ascites and tumors were collected. (A) CKb11, (B) IFN-γ and (C) TNF-α levels in tumor, ascites and serum. (D) CKb11 level in different organs by Elisa. (n = 6, *p < 0.05, **p < 0.01, ***p < 0.001, ****p < 0.0001, n. s. , no significance; One-way ANOVA).

The immunosuppressive TME is a major obstacle to tumor immunogene therapy. Therefore, we tested whether the successful delivery of nanocomposites could reshape the TME in vivo and exert an anti-tumor effect effectively. Firstly, the successful delivery of pCKb11 into mice using our well-designed nanomaterials was proven. Secondly, efficient expression of the delivered immunogene (CKb11) was observed, with the targeted group exhibiting the highest expression level (Fig. 9A). In addition, as shown in Fig. 9B and C, the secretion of inflammatory factors IFN-γ and TNF-α in tumor tissues and the abdominal cavity also increased. At the same time, CKb11 expression in each vital organ was assessed, and no difference was found between each group (Fig. 9D). In the spleen, the expression of CKb11 was increased, which could be attributed to the abundant presence of immune cells in the spleen. However, these inflammatory factors in the serum of mice did not change significantly. This demonstrates that the mice did not have an obvious systemic immune response after treatment.

Moreover, we assessed the alterations of immune cells that had infiltrated the TME following various treatments in vivo. As shown in Figs. S9–S12, the activated CD4^+^ and CD8^+^ T cells labeled with CD69 and IFN-γ were increased in TME after administering pCKb11 and iPD-L1, particularly when using the targeted delivery system. In addition, Fa-PCD/pCKb11/iPD-L1 caused sharp down-regulation of the M2 and up-regulation of M1 macrophages in ascites (Fig. S13). DCs, known as exceptional antigen-presenting cells (APCs), play a crucial role in activating quiescent T cells and are pivotal in initiating, controlling, and sustaining immune reactions. Fa-PCD/pCKb11/iPD-L1 treatment could trigger DC maturation in TME with up-regulated CD11c^+^ CD86^+^ or CD11c^+^ MHCII^+^ cells (Fig. S14). NK cells possess an innate capacity to eliminate cancerous cells without prior stimulation and have a crucial function in the immune surveillance of malignant cells. NK cells have the ability to release cytokines, which can then transform into cytotoxic or effector NK cells in order to stimulate the activation of other immune cells. Treatment with Fa-PCD/pCKb11/iPD-L1 induced the infiltration of NK cells in TME by increased CD49b^+^ CD107a^+^ cell (Fig. S15).

### 3.8 Safety assessment of Fa-PCD/pCKb11/iPD-L1 in vivo

We also assessed the safety of the Fa-PCD/pCKb11/iPD-L1 treatment in mice bearing ovarian cancer. As shown in Fig. S16, the H&E staining of vital organs in all the groups displayed no abnormal histopathological changes. The different treatments were well tolerated by mice without anomalous changes in renal and hepatic function indexes (Fig. S17). In conclusion, the preliminary safety evaluation shows that Fa-PCD/pCKb11/iPD-L1 nanocomposites have no apparent systemic toxicity in vivo.

## 4 Discussion

The immunosuppressive TME facilitates the tumor immune escape, leading to tumor progression [37,38]. Normalizing the immunosuppressive TME by activating anti-tumoral immune cells in TME plays a significant role in immunotherapy [38,39]. Some research has relived the state of immunosuppressive microenvironment through the alleviation of hypoxia and depletion of GSH by a complex drug-delivery system [40]. While we used the CKb11, a member of chemokines, which can effectively activate multiple immune cells, highlighting its potential in recruiting immune cell and facilitating active immunogene therapy [41]. Therefore, this study was to investigate the efficacy and anti-tumor mechanisms of combining pCKb11 mediated immunogene therapy and immune checkpoint inhibitor iPD-L1 using Fa-PCD as an efficient tumor-targeting vector.

The cytokine-based immunotherapy has been extensively studied, while the anticipated efficacy has not been achieved yet [19,42]. In addition to the intricate extraction and purification process of recombinant cytokines, rapid clearance and a short plasma half-life have also been noticed in clinical trials, resulting in diminished therapeutic efficacy [42,43]. Moreover, to reach therapeutically effective concentrations at tumor sites, parenteral administration at high dosages of recombinant cytokines often leads to severe systemic adverse reactions [19]. The rapid development of nanotechnology has provided a promising approach for the application of chemokine-based tumor immunogene therapy [44,45]. The nanoscale vectors for immunogene therapy should prossess high transfection efficiency, tumor-specific uptake, efficient nuclear entry and successful gene expression. Some researches have been utilized the characteristics of TME and designed numerous drug vectors for drug delivery, improving the cancer treatment results [46]. For example, in a research, Yongchao Chu et al. has used the targeting chemotaxis tendency characteristics of neutrophils to the tumor metastasis and devised neutrophil membrane as shell to coat drugs [47]. Similarly, in our study, we have learned the commonly up-regulated expression of the folate receptor on ovarian cancer cells compared to normal tissue cells, the folate receptor has been used as the target for ovarian cancer therapy [35]. In this study, the nanoparticles modified with Fa were prepared for the codelivery of pCKb11 and iPD-L1. Compared to the unmodified nanoparticles, the nanoparticles modified with Fa exhibited enhanced transfection efficiency, leading to successful nuclear uptake by tumor cells and a significant increase in the secretion of CKb11 chemokine. Hence, the Fa-PCD nanoparticles displayed exceptional characteristics as a dependable vehicle for genes.

Immunostimulating cytokines, which can effectively activate immune effector cells within the TME, have emerged as a promising strategy for cancer immunotherapy [48,49]. However, the effects of immunostimulating agents are not usually as good as expected. Cytokine stimulation has been shown to enhance the secretion of IFN-γ from activated immune cells, leading to the induction of PD-L1 expression on tumor cells. This, in turn, hampers T-cell activation and weakens the local anti-tumor immune response [50,51]. Hence, the utilization of immunostimulating treatment along with immune checkpoint inhibitors for the purpose of targeting various immune regulatory pathways holds immense promise in augmenting anti-cancer immune reactions, resulting in significantly amplified therapeutic outcomes. The increased PD-L1 expression on tumor cells co-cultured with activated T lymphocytes after pCKb11 transfection by Fa-PCD was in line with the up-regulation of IFN-γ secretion. This finding motivated us to devise a plan that involves the utilization of iPD-L1 to counteract the suppressive effect of PD-L1 expression and bolster the immune response against tumors. Herein, the combination of pCKb11-based immunogene therapy with iPD-L1 that co-delivered by Fa-PCD nanoparticles exhibited significantly improved anti-tumor efficacy, as indicated by significantly reduced volume of ascites and abdominal dissemination compared to pCKb11 or iPD-L1 alone with Fa-PCD nanoparticles. This combined treatment significantly enhanced the anti-tumor immune responses with increased activation of immune cells. Moreover, high concentrations of CKb11, TNF-α and IFN-γ were detected in tumors and ascites without significant changes in CKb11, TNF-α, IFN-γ levels in the serum, indicating locally activated anti-tumor immunity without obvious systemic inflammation responses. This further supported the benefits of combination therapy. In summary, activating tumor local immunity while suppressing tumor immune escape by co-delivering pCKb11 and iPD-L1 in Fa-PCD nanoparticles in our study has achieved robust therapeutic efficacy without noticeable adverse effects.

The activation and infiltration of immune cells in the TME is the primary approach for achieving anti-tumor immunotherapy [52,53]. The present research demonstrated that the combined therapy effectively improved the immune response against tumors by promoting the activation of T cells, augmenting the presence of mature dendritic cells within the tumor, and reducing the infiltration of M2-polarized macrophages. Activated lymphocytes can secrete cytokines, directly kill target cells, and facilitate immune responses [54]. This study shows that combined therapy of pCKb11 and iPD-L1 promotes lymphocyte proliferation， activation，and secretion of TNF-α and IFN-γ. The abundant secretion of IFN-γleads to high expression of PD-L1, highlighting the potential of adding iPD-L1 into the combinational strategy. In addition, DCs are the most potent professional antigen-presenting cells, which act as messengers, transmitting antigen information to T cells, and activating them [55,56]. A small amount of DCs can trigger a powerful T-cell response. Mature DCs in the TME were increased after treatment with combined therapy of pCKb11 and iPD-L1, which surveil and kill tumors by recognizing tumor-specific antigens and presenting their signals to killer T cells. As one of the most recruited immune cells in the TME, the majority of TAMs are M2 phenotype which is closely linked to tumor progression and poor prognosis [57,58]. The M2-polarized macrophages have the ability to enhance the growth and formation of blood vessels in tumor tissues, sustain the characteristics of cancer stem cells, and hinder the immune response against tumors by producing PD-L1 or immunosuppressive substances that suppress the activity of cytotoxic T lymphocytes (CTLs) [[59], [60], [61]]. Due to the significant impact of TAMs on inhibiting tumor immunity and advancing tumor advancement, there has been considerable fascination with approaches aimed at TAMs. These approaches primarily involve depleting TAMs or modifying TAM polarization [62]. Promoting the transformation of TAMs into the tumoricidal phenotype could potentially enhance immunotherapy efficacy, instead of eliminating TAMs and sacrificing their immunostimulatory function as antigen-presenting cells or phagocytes within tumor tissues. In this study, we showed the co-delivery of pCKb11 and iPD-L1 by Fa-PCD nanoparticles could effectively inhibit M2-polarization. Fa-PCD can induce the activation of transcript factors such as p65, STAT1, IRF5, IRF3, and iNOS while suppressing the activation of IRF4 and PPAR-r. This leads to the repolarization of macrophages from an M2 phenotype to an anti-tumoral M1 phenotype when co-administered with pCKb11 and iPD-L1. Therefore, the co-delivery of pCKb11 and iPD-L1 by Fa-PCD could efficaciously reshape the TME via inhibiting the M2-polarizaiton of TAMs, which may be an anti-tumor strategy with excellent application prospects and exploring values.

The primary challenge in current immunotherapy lies in striking a balance between an effective therapeutic dose and the occurrence of immune-related adverse events (irAEs) [63,64]. While obtaining an effective dose, a high concentration of cytokines outside the tumor site usually leads to severe treatment-related toxicity. Therefore, the immunogene therapy of pCKb11 delivered by Fa-modified nanocarriers can effectively target tumor cells to secrete the chemokine CKb11. The tumor site experiences a strong accumulation of pCKb11, which efficiently triggers the activation of different immune cells and modifies the immunosuppressive TME, all while avoiding a systemic immune response. However, locally activated immunity leads to a substantial secretion of immune-stimulating cytokines, such as IFN-γ. IFN-γ is a double-edged sword that inhibits tumor growth by regulating immune response, promoting cell apoptosis and inhibiting angiogenesis [65]. On the other hand, it can lead to up-regulation of PD-L1 expression at the tumor cells to promote tumor cell immune escape [60]. Therefore, adding the immune checkpoint inhibitor can exert a synergistic therapeutic effect with pCKb11 immunogene therapy. Therefore, our strategy using the Fa-modified delivery system of pCKb11 combined with iPD-L1 to tumor sites to activate multiple immune regulatory pathways has great potential for synergistic improvement of therapeutic efficacy.

## 5 Conclusion

The novel nanoparticles targeting folic receptor could efficiently and safely deliver pDNA encoding CKb11 and small molecular inhibitor that blocks PD-1/PD-L1 interactions to tumor sites and fight against cancer through remodeling the immunosuppressive TME by activating T lymphocytes, inhibiting macrophages towards type M2, and promoting DCs maturation in TME. Thus, using the nanocomposites to achieve tumor-targeting co-delivery of immunostimulating chemokine and immune checkpoint inhibitor processes great potential for translation to clinic immunogene therapy.

Ethics approval and consent to participate

All animal experiments were carried out in accordance with the regulations of the Animal Experiment Ethics Committee of the State Key Laboratory of Biotherapy, Sichuan University.

## CRediT authorship contribution statement

**Wen Nie:** Methodology, Investigation, Funding acquisition. **Yihong He:** Project administration, Methodology, Investigation. **Xue Mi:** Methodology, Investigation, Formal analysis. **Shi He:** Project administration, Methodology, Investigation. **Jing Chen:** Methodology. **Yunchu Zhang:** Methodology, Investigation. **Bilan Wang:** Project administration, Methodology, Funding acquisition. **Songping Zheng:** Writing – review & editing, Investigation, Funding acquisition. **Zhiyong Qian:** Writing – review & editing, Writing – original draft. **Xiang Gao:** Writing – review & editing, Project administration, Funding acquisition.

## Declaration of competing interest

The authors declare that they have no known competing financial interests or personal relationships that could have appeared to influence the work reported in this paper.
