# Supplementary material for: Immunostimulatory CKb11 gene combined with immune checkpoint PD-1/PD-L1 blockade activates immune response and simultaneously overcomes the immunosuppression of cancer
Source: Bioact Mater. 2024 May 23;39:239–54. doi: 10.1016/j.bioactmat.2024.05.014 (PMC11145080; doi:10.1016/j.bioactmat.2024.05.014)
Supplement: Multimedia component 1 [file mmc1.docx]

**Materials and methods**

***Synthesis of Fa-PEG-PCL and MPEG-PCL***

HOOC-PEG_2000_-OH was purchased from Ponsure Biotechnology (Shanghai, China). L-lactide and MPEG2000 were bought from Sigma-Aldrich co. Fa was purchased from Shanghai Macklin Biochemical Technology Co., Ltd. Briefly, MPEG-PCL was synthesized by ring-opening polymerization of ε-caprolactone (ε-CL) using MPEG_2000_-OH as an initiator. Fa-PEG-PCL was synthesized by ring-opening polymerization of ε-caprolactone (ε-CL) using HOOC-PEG_2000_-OH as an initiator followed by two-step condensation reaction with Fa. The structure and degree of polymerization of the co-polymers were characterized by hydrogen nuclear magnetic resonance spectroscopy (^1^H-NMR, Bruker, Germany).

***Safety evaluation***

The blood samples of the mice were collected and used to analyze some important serological biochemical indicators using an automatic analyzer (Hitachi High-Technologies Crop., Japan). Moreover, the organ tissues, including the heart, liver, spleen, lungs and kidneys of the tumor-bearing mice in each group, were also collected, fixed and sectioned for H&E staining to assess the organ-specific toxicity.

***Therapeutic efficacy evaluation***

The next day after completing the treatment, mice were euthanized, and the tumor nodules, ascites, blood and vital organs were collected. Mice and tumors were weighed. Corresponding cells were collected to perform FCM analysis of the TME. Supernatants of ascites, tumor tissue lysis product and serum were analyzed by ELISA assay. Tumors and organs were fixed, embedded, and H&E or immunohistochemical stained for histopathological examination. Tumor cell proliferation was determined by Ki67 staining, and microvessels was evaluated by CD31 staining according to the immunohistochemistry staining protocols. The number of CD31-positive vessels and proportion of Ki67-positive cells were counted and analyzed with five randomized fields.

**Table S1.** Sequences of PCR primers used in this study.

| PCR primers | |
| --- | --- |
| NOS2 | Forward: 5’-CGAAACGCTTCACTTCCAA-3’  Reverse: 5’-TGAGCCTATATTGCTGTGGCT-3’ |
| IL-12 | Forward:5’-CTGGAACTACACAAGAACGAGAG-3’  Reverse:5’-GGCACAGGGTCATCATCAAA-3’ |
| Citta | Forward:5’-CTCAGCCACCTTCCCTCA-3’  Reverse:5’-CAGTGATGTTGTTTTGGGACA-3’ |
| TNF-α | Forward:5’-TCTCAGCCTCTTCTCATTCCTGCT-3’  Reverse:5’-AGAACTGATGAGAGGGAGGCCATT-3’ |
| IFN-γ | Forward:5’-GGATGCATTCATGAGTATTGC-3’  Reverse:5’-CCTTTTCCGCTTCCTGAGG-3’ |
| IRF5 | Forward:5’-AATACCCCACCACCTTTTGA-3’  Reverse:5’-TTGAGATCCGGGTTTGAGAT-3’ |
| CXCL9 | Forward: 5’-GAGGAACCCTAGTGATAAGGA-3’  Reverse: 5’-CCTTGAACGACGACGACTTTG-3’ |
| CXCL10 | Forward:5’-GGAAGCCTCCCCATCAGCACC-3’  Reverse:5’-AGACAGGCTCTCTGCTGTCCA-3’ |
| Fizz1 | Forward:5’-CCTGCTGGGATGACTGCTA-3’  Reverse:5’-TGGGTTCTCCACCTCTTCAT-3’ |
| IL-6 | Forward:5’-ACAAGTCGGAGGCTTAATTACACAT-3’  Reverse:5’- TTGCCATTGCACAACTCTTTTC-3’ |
| IRF4 | Forward:5’-AATGGGAAACTCCGACAGTG-3’  Reverse:5’-TAGGAGGATCTGGCTTGTCG-3’ |
| YM-1 | Forward:5’-GCCACTGAGGTCTGGGATGC-3’  Reverse:5’-TCCTTGAGCCACTGAGCCTTC-3’ |
| MRC-1 | Forward:5’-AAGGCTATCCTGGTGGAAGAA-3’  Reverse:5’-AGGGAAGGGTCAGTCTGTGTT-3’ |
| GAPDH | Forward:5’-GGAGCGAGATCCCTCCAAAAT-3’  Reverse:5’-GGCTGTTGTCATACTTCTCATGG-3’ |

**Table** **S2**. Information of flow cytometry antibodies used in this study

| **Antibodies** | | |
| --- | --- | --- |
| FITC anti-mouse CD4 | Biolegend | Cat. 130308 |
| PE anti-mouse CD69 | Biolegend | Cat. 104508 |
| FITC anti-mouse CD8a | Biolegend | Cat. 100804 |
| PE-Cy7 anti-mouse CD8a | Biolegend | Cat. 162312 |
| Percp-Cy5.5 anti-mouse CD4 | Biolegend | Cat. 100434 |
| APC anti-mouse IFN-γ | Biolegend | Cat. 113606 |
| APC-Cy7 anti-mouse CD45 | Biolegend | Cat. 103116 |
| FITC anti-mouse/human CD11b | Biolegend | Cat. 101206 |
| PE anti-mouse F4/80 | Biolegend | Cat. 111604 |
| APC anti-mouse CD206 | Biolegend | Cat. 141708 |
| PE-Cy5 anti-mouse CD11c | Biolegend | Cat. 117316 |
| APC hamster anti-mouse CD80 | BD Biosciences | Cat. 560016 |
| APC anti-mouse I-A/I-E | Biolegend | Cat. 107614 |
| FITC anti-mouse I-A/I-E | Biolegend | Cat. 107606 |
| PE anti-mouse CD86 | Biolegend | Cat. 159204 |
| FITC rat anti-mouse CD49b | BD Biosciences | Cat. 553857 |
| PE anti-mouse CD107a (LAMP-1) | Biolegend | Cat. 121612 |
| PER rat anti-mouse CD274(PD-L1) | BD Biosciences | Cat. 568085 |





**Fig. S1** The synthetic procedure of MPEG-PCL





**Fig. S2** The synthetic procedure of Fa-PEG-PCL


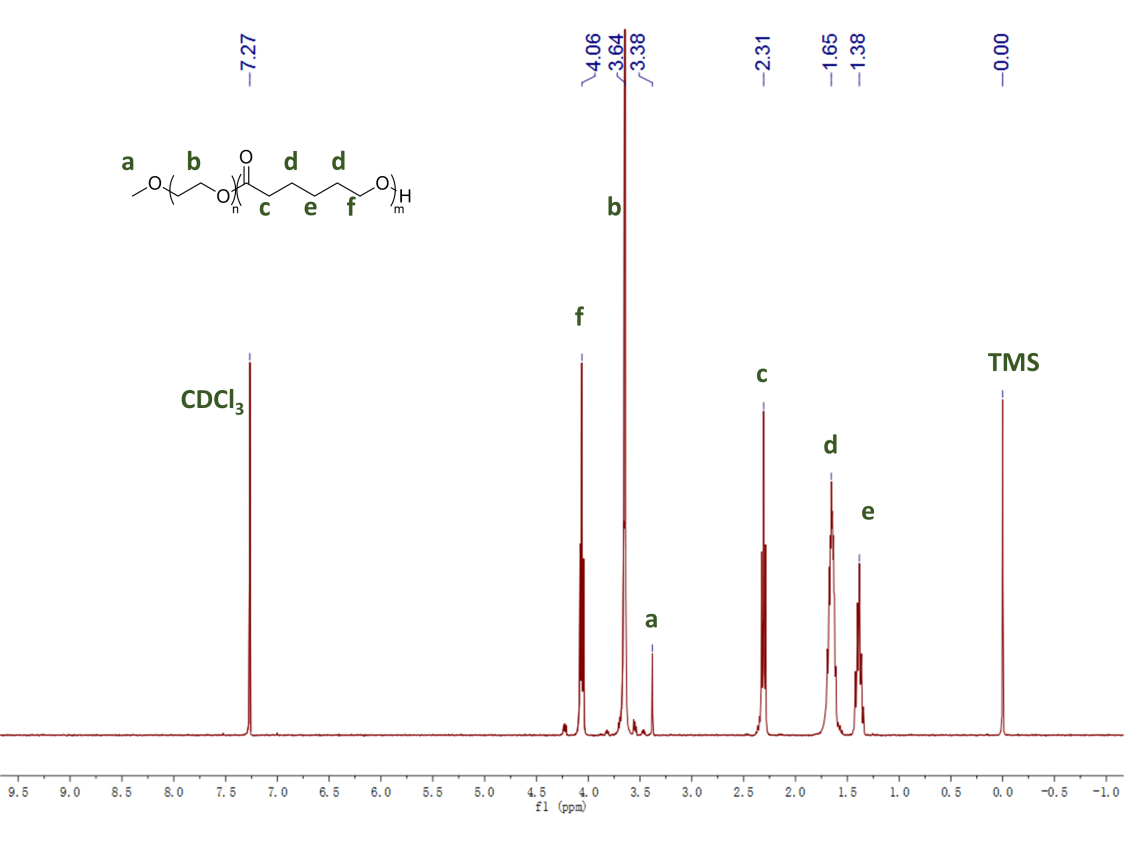


**Fig. S3** The ^1^H NMR spectrum of polymer MPEG-PCL in CDCl_3_ (400 MHz, CDCl_3_, 25℃)


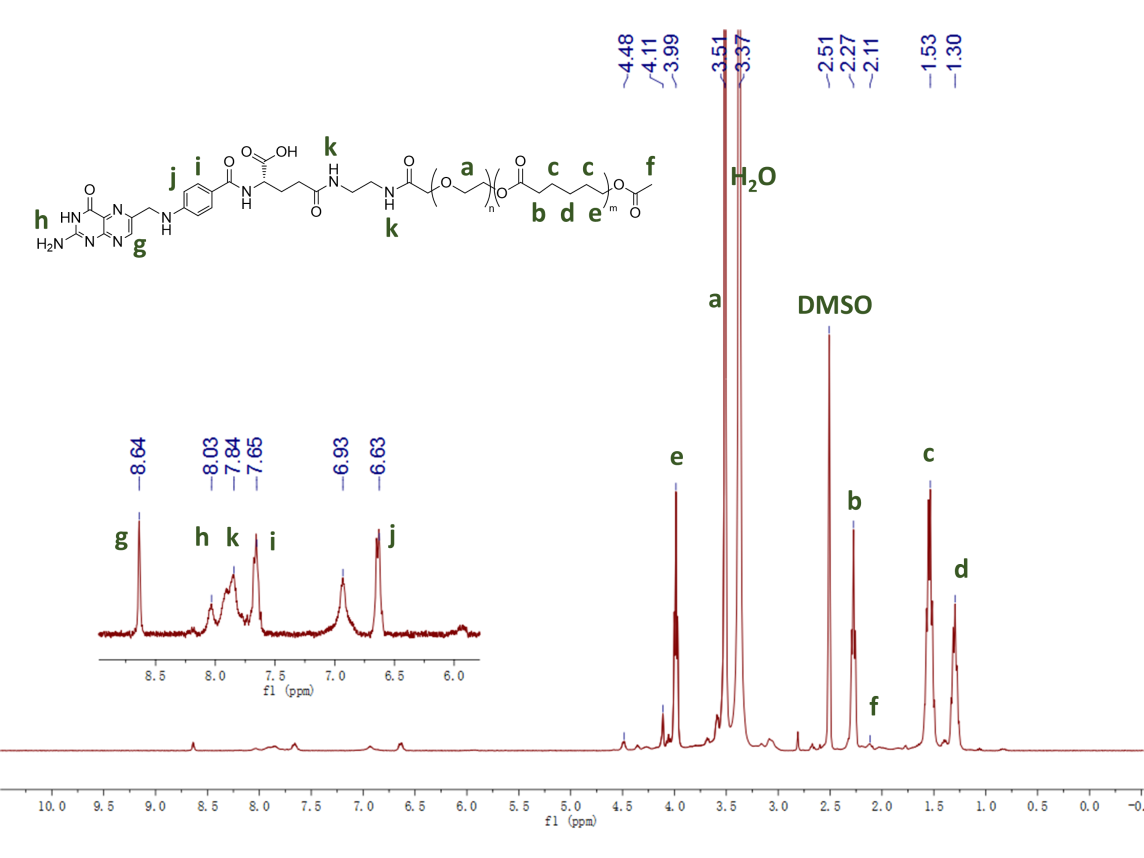


**Fig. S4** The ^1^H NMR spectrum of polymer Fa-PEG-PCL in DMSO-D_6_ (400 MHz, DMSO-D_6_, 25℃)


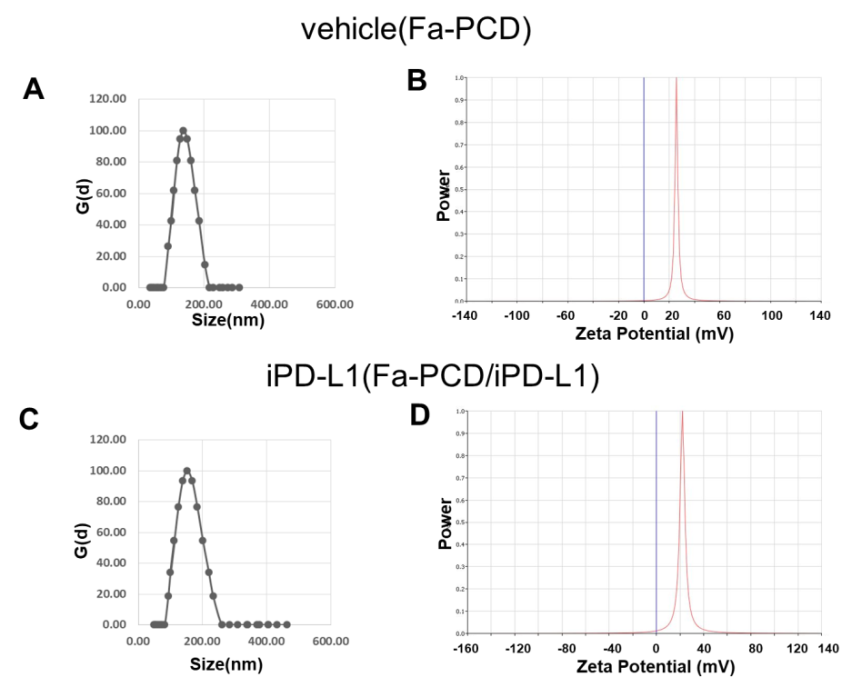


**Fig. S5. Characteristics of control nanocomposites.** Size and *zeta* potential of vehicle (Fa-PCD) (A, B) and Fa-PCD/iPD-L1 (C, D). (n=3)


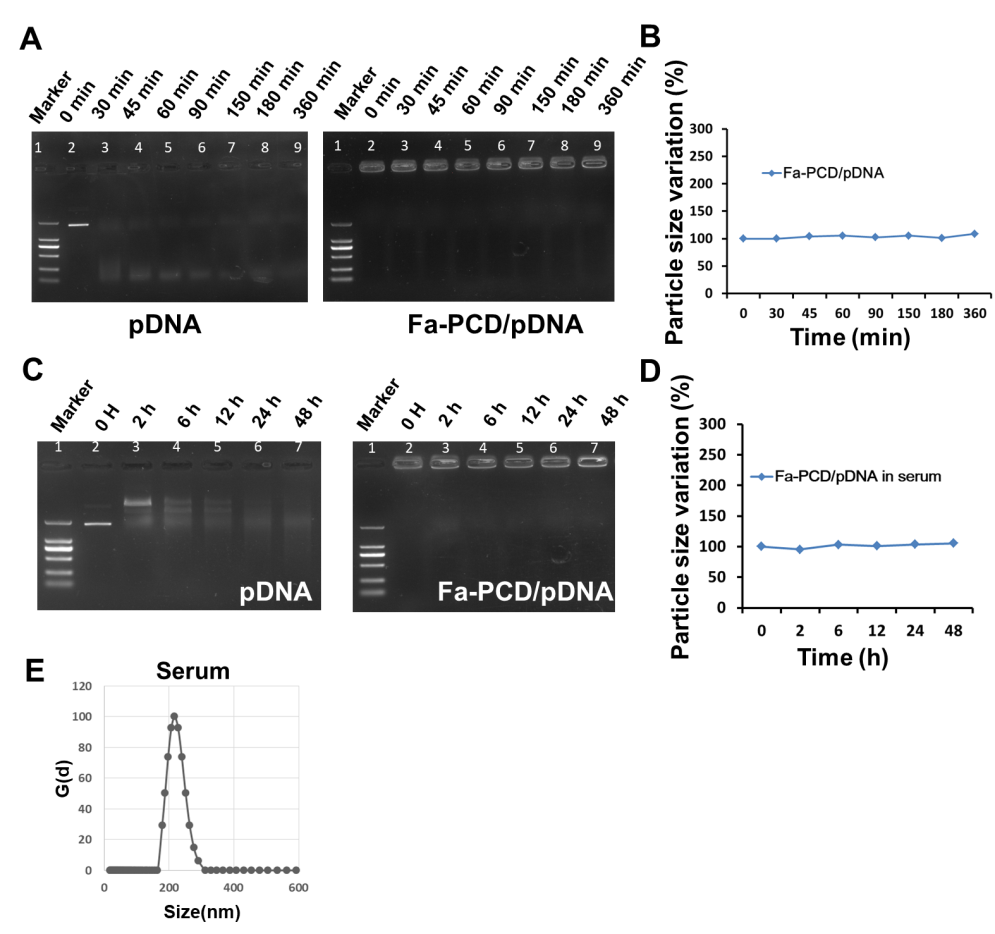


**Fig. S6 Fa-PCD nanoparticles resist nuclease degradation.** pDNA and Fa-PCD/pDNA nanoparticles were incubated in 1640 media with DNase I (1U/ml) for different time (A, B). (A) Agarose gel electrophoresis of pDNA and Fa-PCD/pDNA. (B) Particle size variation of Fa-PCD/pDNA at different time. pDNA and Fa-PCD/pDNA nanoparticles were incubated in serum (fresh 1640 media with 10% FBS) for different time (C, D). (C) Agarose gel electrophoresis of pDNA and Fa-PCD/pDNA. (D) Particle size variation of Fa-PCD/pDNA at different time. (E) Size of F-PCD/pDNA nanoparticles in serum after 48 h.(n=3)


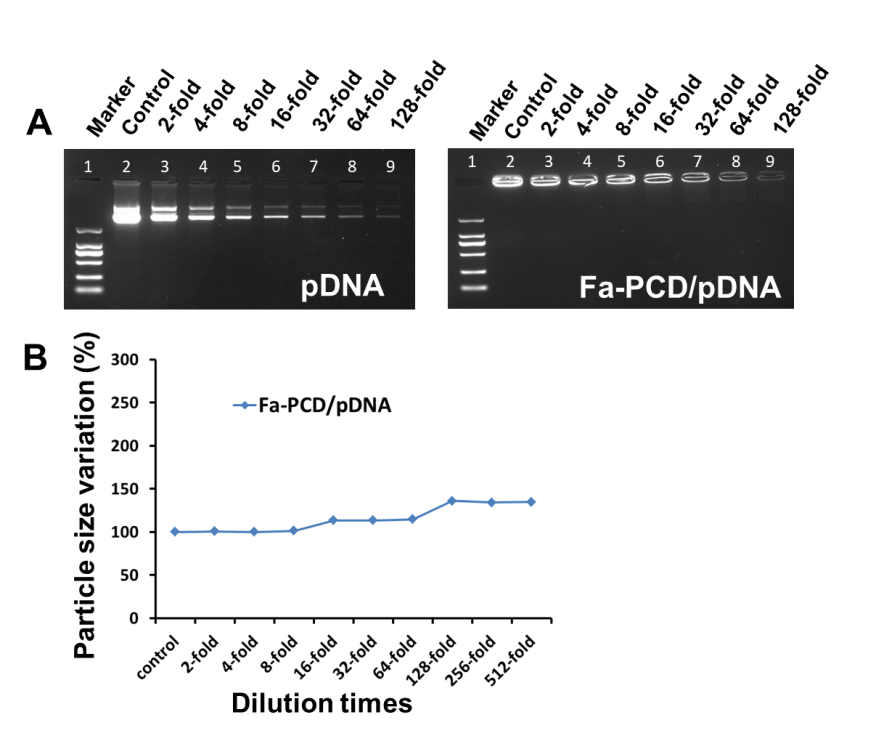


**Fig. S7 Dilution stability of Fa-PCD/pDNA.** (A) Agarose gel electrophoresis retardation assay for detection of loosening or disassembly of DNA polyplex in a serial dilution process and (B) the size variation of Fa-PCD/pDNA at a serial dilution. (n=3)


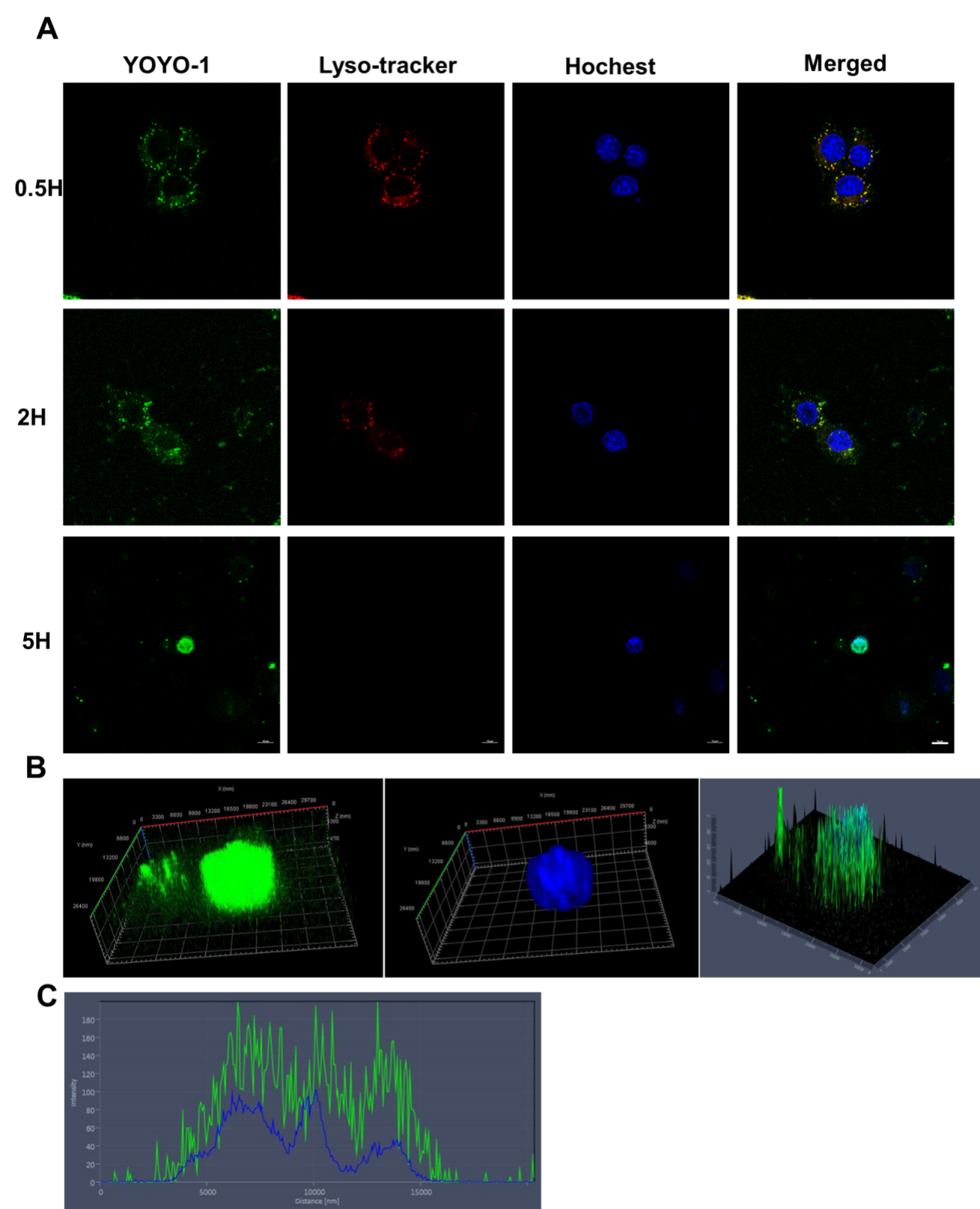


**Fig. S8** Confocal images of ID8 cells treated with Fa-PCD/pCKb11. pCKb11 was labeled with YOYO-1, lysosomes were stained with Lyso-tracker and the nuclei were stained with Hoechst 33342. (A) Images of cells were taken at 0.5, 2 and 5 h, respectively (scale bar: 10 μm). (B) Left, middle and right, 3-dimensional images taken at 5 h. (C) Analysis of fluorescence intensity in cross-section of cells post 5 h.


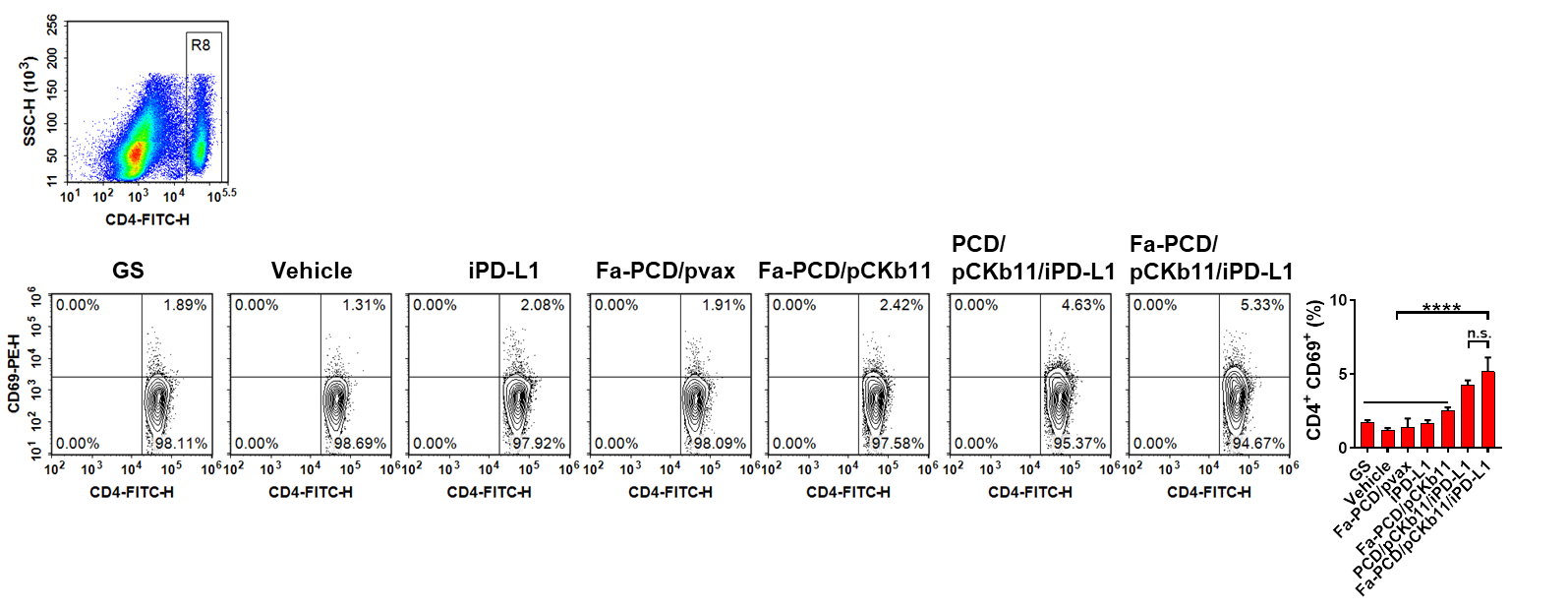


**Fig. S9** The subsets of CD4^+^CD69^+^ lymphocytes in the peritoneal lavage fluid of mice with abdominal metastasis of ovarian cancer were measured by FCM. (n = 3, ****p < 0.0001, n.s. , no significance; One-way ANOVA).


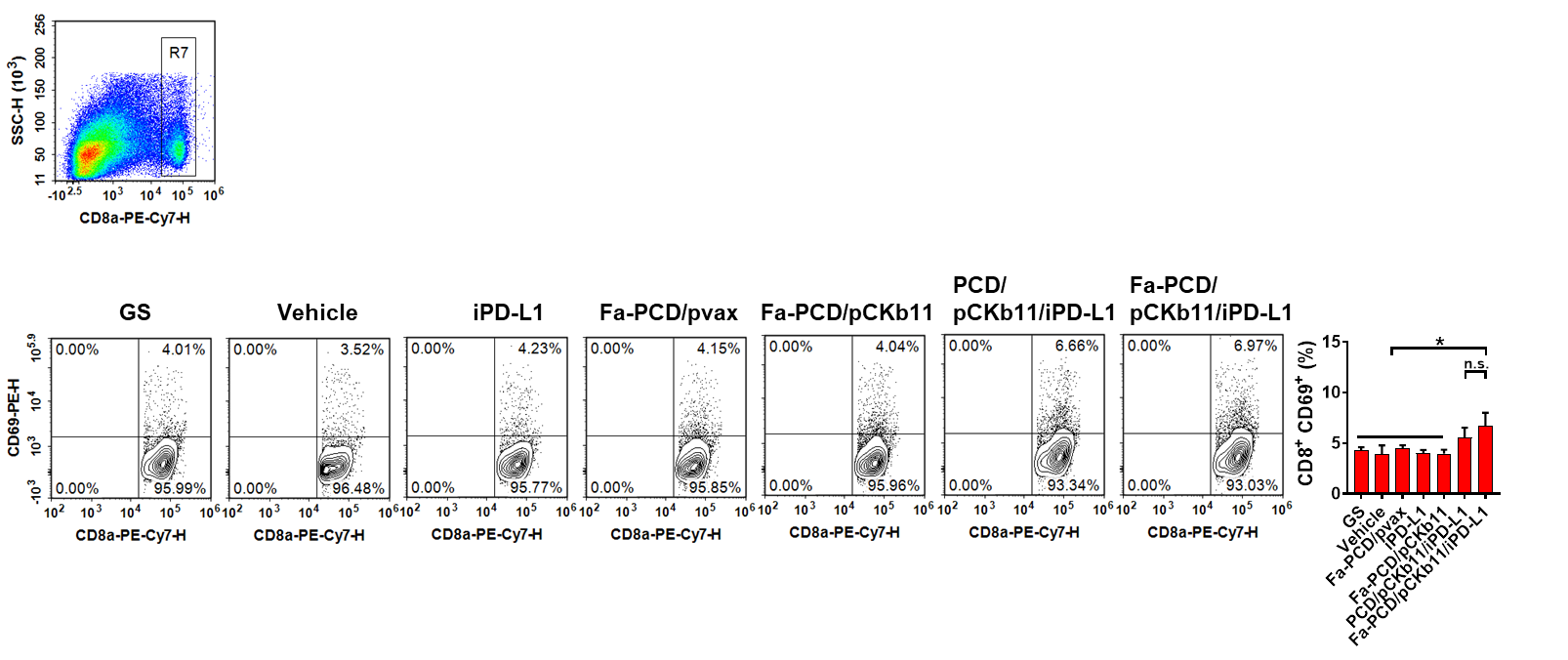


**Fig. S10** The subsets of CD8^+^CD69^+^ lymphocytes in the peritoneal lavage fluid of mice with abdominal metastasis of ovarian cancer measured by FCM. (n = 3, *p < 0.05, n.s. , no significance; One-way ANOVA).


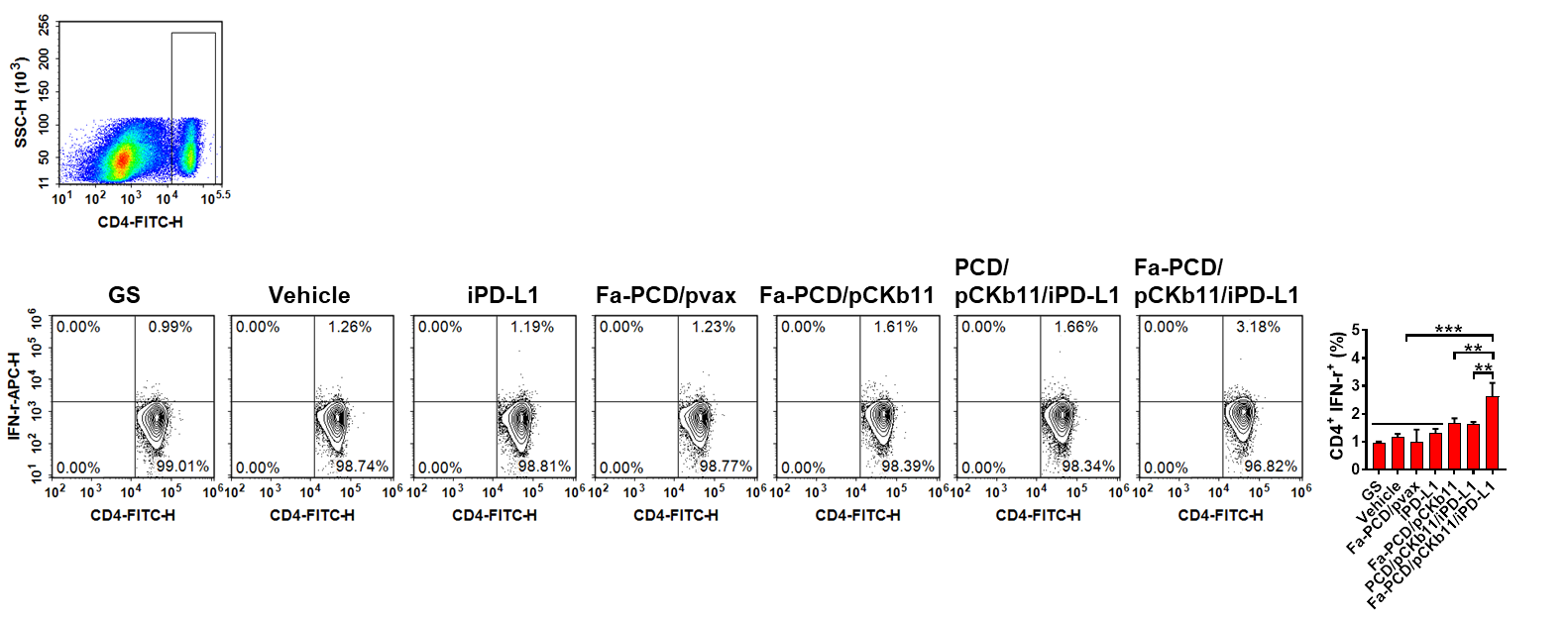


**Fig. S11** The subsets of CD4^+^IFN-γ^+^ lymphocytes in the peritoneal lavage fluid of mice with abdominal metastasis of ovarian cancer measured by FCM. (n = 3, **p < 0.01, ***p < 0.001, One-way ANOVA).


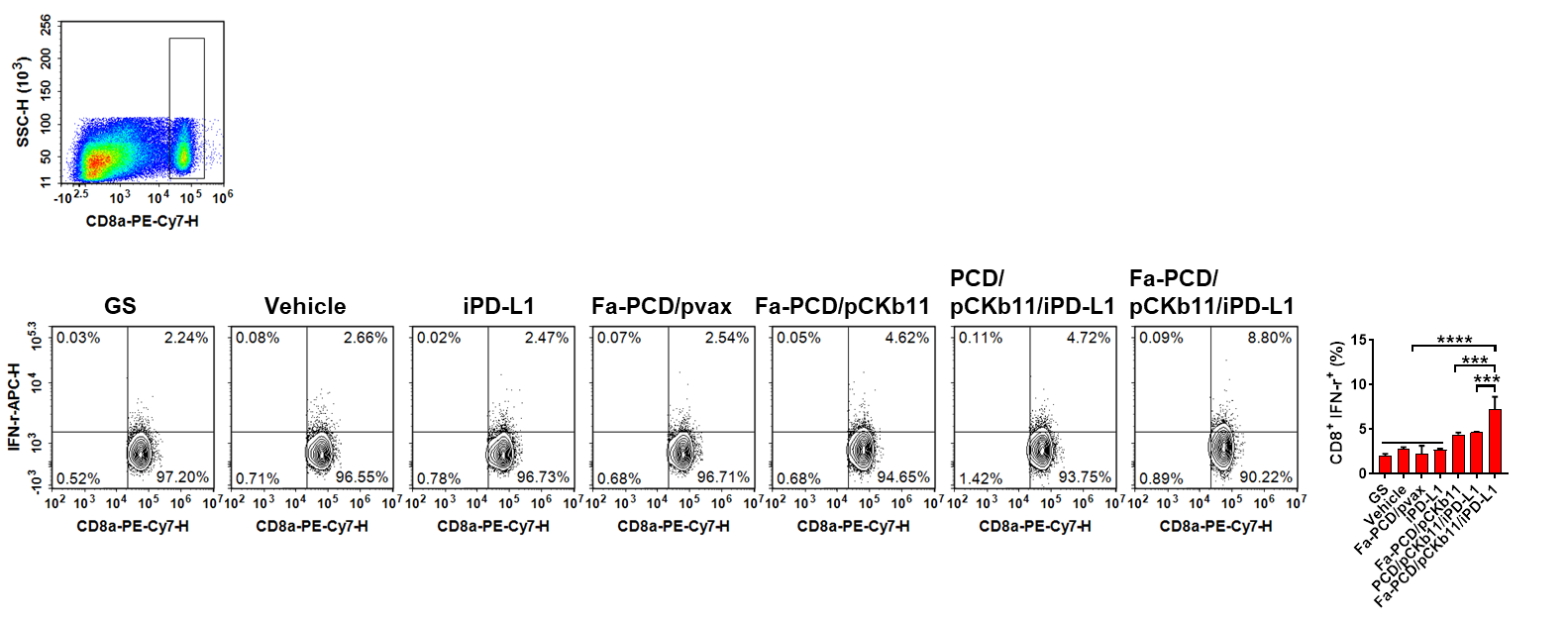


**Fig. S12** The subsets of CD8^+^IFN-γ^+^ lymphocytes in the peritoneal lavage fluid of mice with abdominal metastasis of ovarian cancer measured by FCM. (n = 3, ***p < 0.001, ****p < 0.0001, One-way ANOVA).


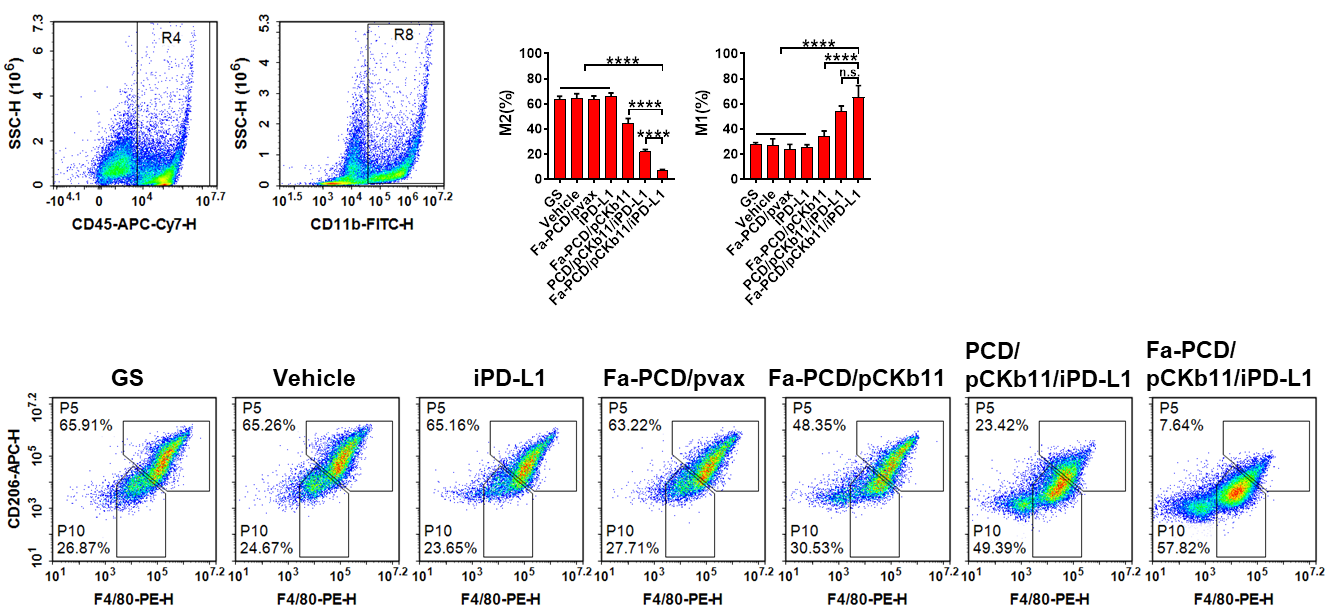


**Fig. S13** The peritoneal lavage fluids of mice collected to analyze the polarization status of macrophages by FCM. M1 cells were defined by CD45^+^CD11b^+^F4/80^+^CD206^-^. M2 cells were defined by CD45^+^CD11b^+^F4/80^+^CD206^+^. (n = 3, ****p < 0.0001, n.s. , no significance; One-way ANOVA).


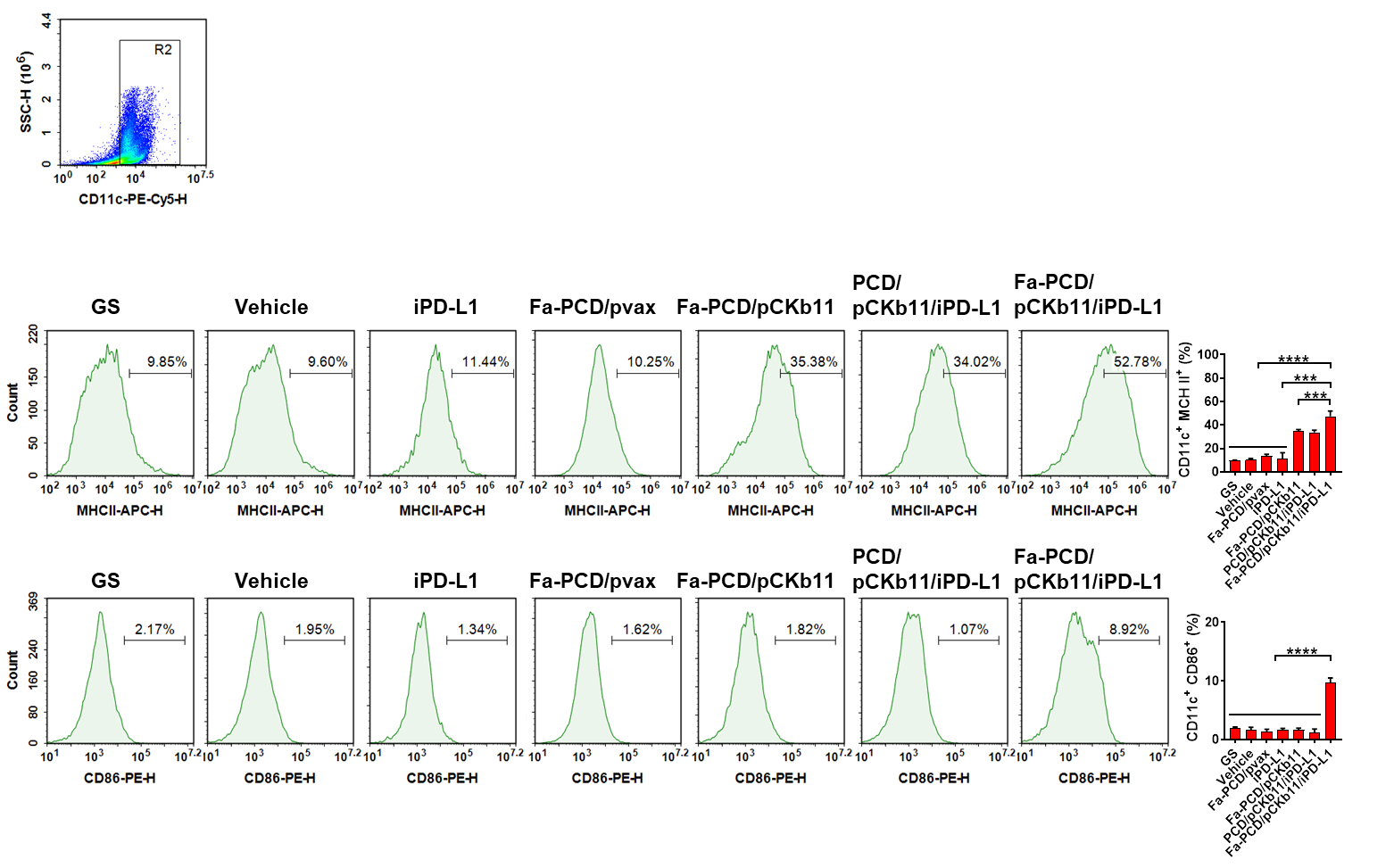


**Fig. S14** The maturation of tumor-infiltrating DCs defined by CD11c^+^MCH II^+^ or CD11c^+^CD86^+^ assessed by FCM. (n = 3, ***p < 0.001, ****p < 0.0001; One-way ANOVA).


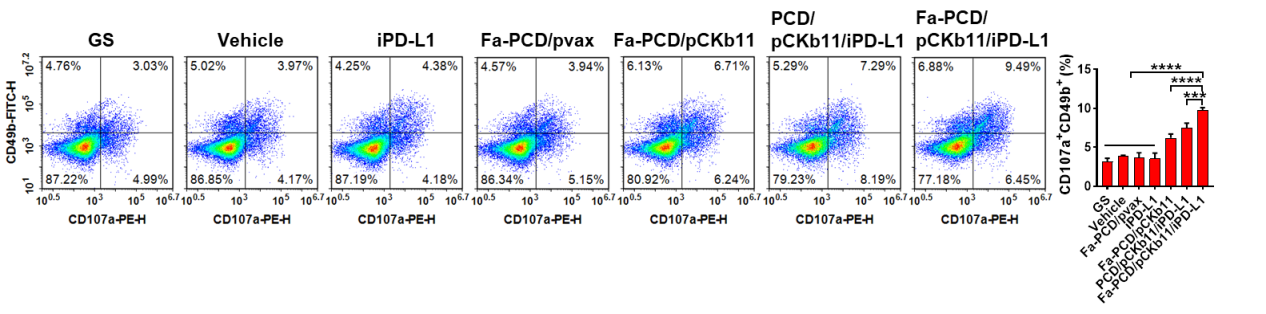


**Fig. S15** The proportion of tumor-infiltrating NK cells defined by CD107a^+^CD49b^+^ evaluated by FCM. (n = 3, ***p < 0.001, ****p < 0.0001; One-way ANOVA).


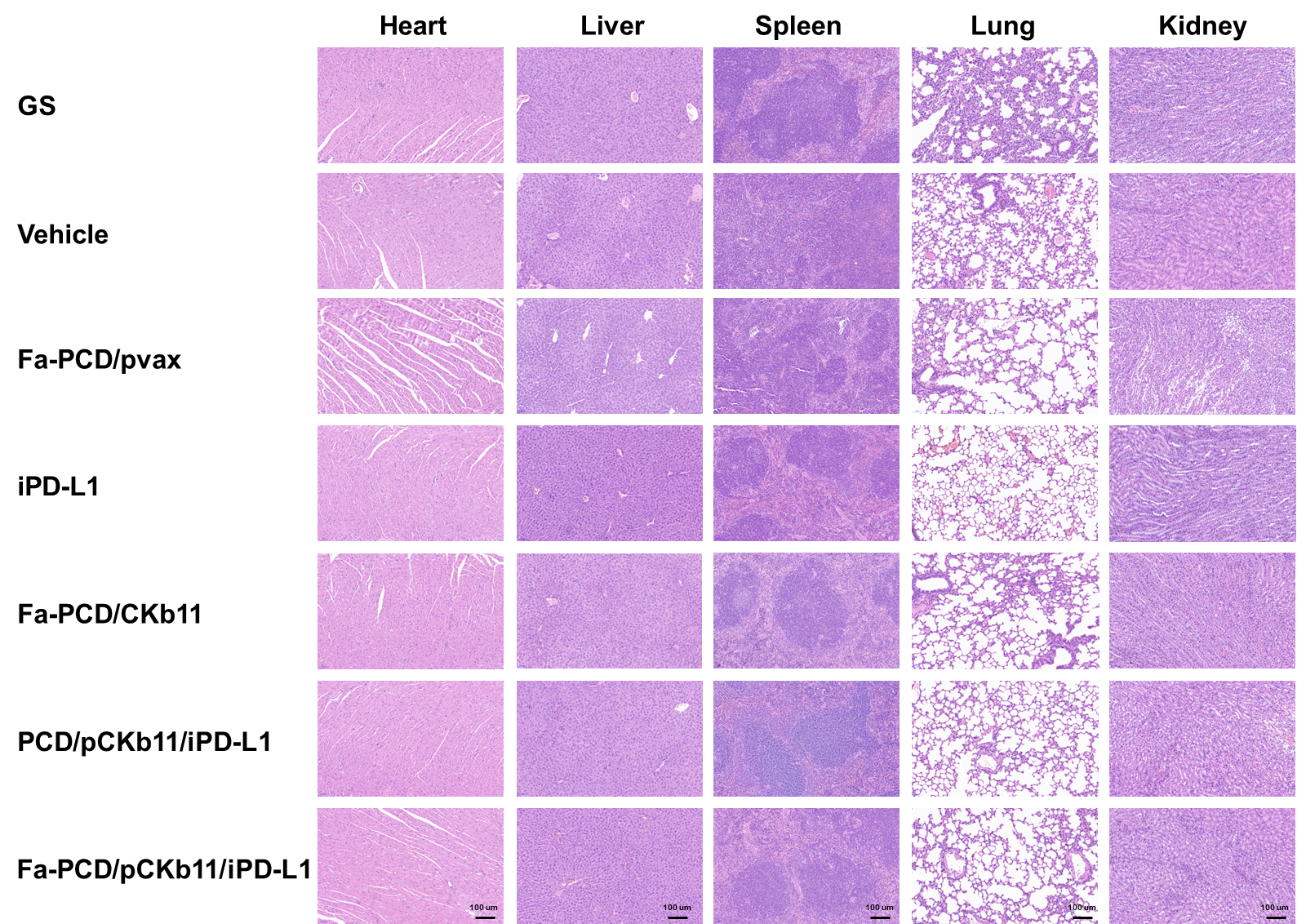


**Fig. S16** Vital organs of heart, liver, spleen, lung and kidney were collected from mice after treatment and stained with H&E staining for histology examination (scale bar, 100 μm; n = 3).


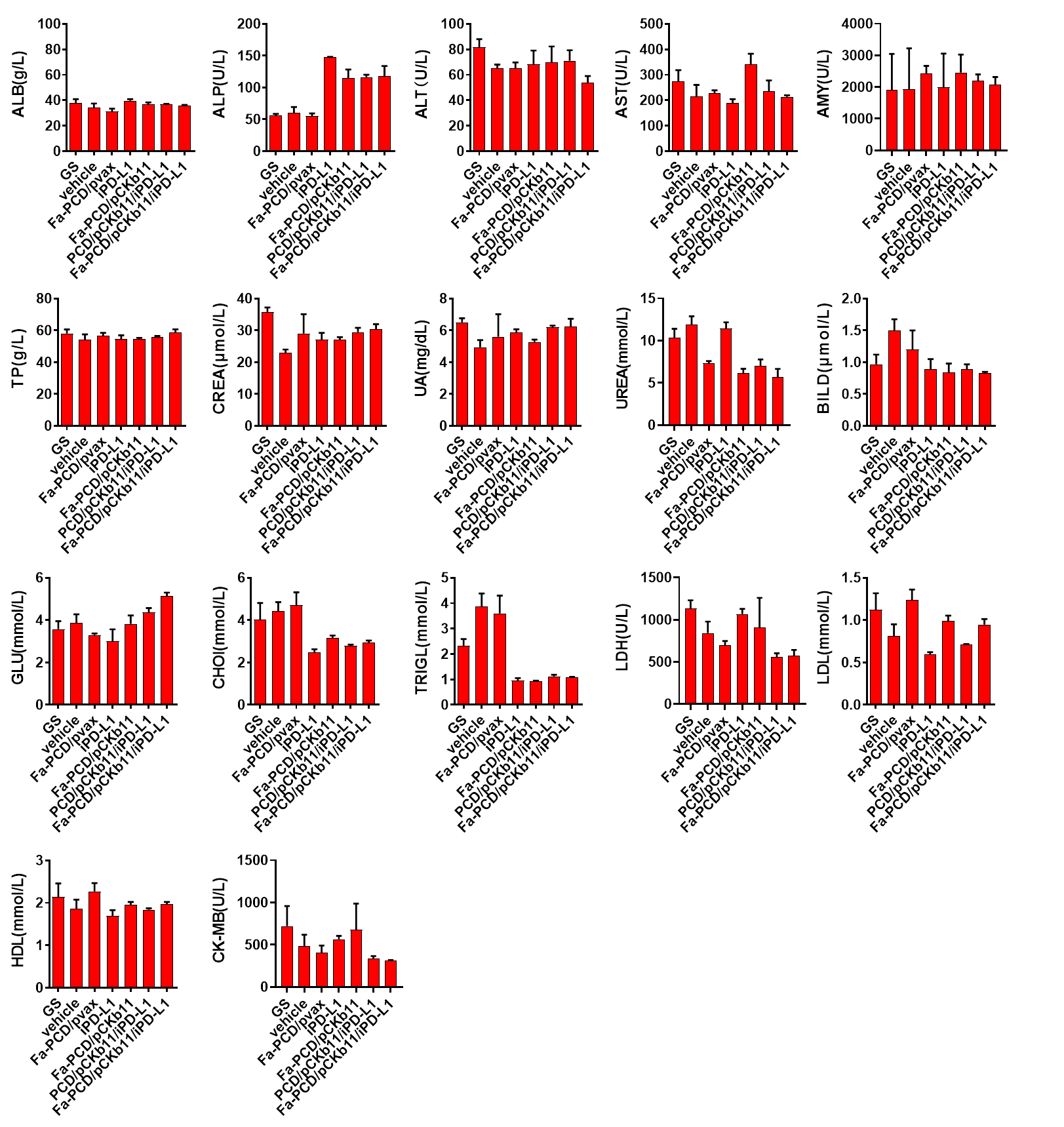


**Fig. S17** Serological biochemical analysis. (n = 6)
